# A Mechanistic Study of Bio‐Based Nanotemplated Carbon Nanofibers Derived From Water Processable Lignin Blends for Sustainable Energy Storage Applications

**DOI:** 10.1002/adma.72508

**Published:** 2026-02-11

**Authors:** Judith Miralda‐Jalle, Jamal El Haskouri, Shayon Bhattacharya, Anne Beaucamp, Tadhg Kennedy, Mario Culebras, Maurice N. Collins

**Affiliations:** ^1^ School of Engineering University of Limerick Limerick Ireland; ^2^ Bernal Institute University of Limerick Limerick Ireland; ^3^ Institute of Material Science Universitat de València Valencia Spain; ^4^ School of Biological Sciences University of Limerick Limerick Ireland; ^5^ Department of Chemical Sciences University of Limerick Limerick Ireland

**Keywords:** batteries, hard carbon, lignin, Na ion

## Abstract

This article studies the process for obtaining sustainable and environmentally friendly, carbon nanofibers via an electrospinning process using water without the need for organic solvents or synthetic polymers/binders. Lignosulfonate, gelatin, and alginate are selected for water solubility, ability to produce templated carbon nanostructures and for sustainability. Gelatin and alginate are sacrificial templates during thermal processing, allowing the production of engineered high surface area hollow nanostructures. A mechanistic study is performed to elucidate the relationship between carbon hybridization and electrochemical performance. The as‐spun carbon materials were further characterized for potential applications in sodium‐ion batteries.

## Graphical abstract

As‐spun carbon materials produced from Lignosulfonate, gelatin, and alginate, selected for water solubility, and ability to produce templated sustainable carbon nanostructures. Gelatin and alginate are sacrificial during thermal processing, allowing the production of engineered high surface area nanostructures, which are further characterized for potential applications in sodium‐ion batteries.A mechanistic study elucidates the relationship between carbon hybridization and electrochemical performance.

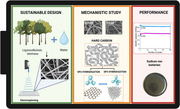

## 1 Introduction

Given the current context of climate change, there is a key necessity to develop sustainable materials for new energy storage systems and implement sustainable design when developing these new materials. With the limited global reserves of lithium, there is a shift toward diversifying battery technologies, driving interest in more sustainable and abundant alternatives, such as sodium‐ion batteries. Hard carbon is a widely investigated anode material for sodium‐ion batteries due to its ability to accommodate sodium ions through both insertion and adsorption mechanisms, excellent cycling stability, and its precursors' abundance and low cost [1, 2]. Hard carbon is a non‐graphitizable carbon that consists of a composite of sp^2^ and sp^3^ hybridized carbon [3, 4, 5, 6]. Carbon bonded through sp^2^ hybridization has three sp^2^ orbitals in trigonal geometry (120) and an unhybridized p‐orbital. This allows sp^2^ carbon to have three identical shapes, sizes, and energies of binding *σ*‐bonds and one *π*‐bond from the delocalized electrons. While Sp^3^‐bonded carbon presents four sp^3^ orbitals, identical in size, shape, and energy. Sp^3^ carbon only forms *σ*‐bonds, which gives sp^2^ hybridized carbon a planar distribution thanks to the trigonal geometry of the *σ*‐bonds, see Figure S1 [5]. In addition, the delocalized electron in the sp^2^ hybridization gives this carbon higher conductivity and a pseudo‐graphitic conformation [6].

The conventional processes to synthetize hard carbon rely on fossil‐based carbon precursors such as polyacrylonitrile (PAN), polypyrrole (PPy), and phenolic resins which allow precise control of their synthesis and tailored nanostructures [7]. Despite the advantages in both function and facile manufacture of synthetic polymers, they are unsustainable and expensive. In contrast, the aromatic structure of lignin, rich in carbon, makes this natural polymer a sustainable carbon precursor [8]. This non‐regular organic polymer made of phenol sub‐units, is the second most abundant biomass polymer, producing over 80–90 million tons per annum of kraft lignin from wood pulping processes [9, 10, 11] and, while it is still considered a by‐product, its application as a sustainable carbon‐precursor could revalorize this natural polymer.

To obtain a lignin product for energy storage applications, electrospinning is a common technique, used to obtain nanostructured materials that show good electrochemical performance [12, 13]. The working principle of electrospinning is based on the application of a high voltage to an electrically responsive solution, which results in the formation of a conical droplet called the Taylor cone [14]. When the electrostatic forces are stronger than the surface tension forces, a jet is ejected from the conical structure and collected into a grounded plate [13, 15, 16]. This process enables the creation of custom materials with hierarchical structures. When electrospinning natural polymers, usually organic solvents or acids such as Dimethylformamide (DMF) and boric acid are used [17, 18, 19, 20, 21, 22, 23], in high concentrations 60%–90% v/v. However, these solvents are unsustainable, hazardous, and can be toxic to humans and the environment.

Hence, there is a requirement to increase the sustainability of electrospinning of natural polymers to revalorize waste such as lignin into high‐end products. The transition from an organic/acid‐based system to an aqueous‐based system is key and so far, has proved challenging for lignin‐based materials; lignosulfonate allows processing in water. However, challenges associated with water in electrospinning remain. With rapid evaporation influencing fiber morphology and chain alignment. While high conductivity of water impacts stretching of the solution into fibers, and high surface tensions lead to decreased specific area of the jet, resulting in congregation and blockages [14, 15]. Polyelectrolytes such as lignosulfonates stabilize interfaces by decreasing surface tension to mitigate this.

In this study, hard carbon is obtained from a sustainable carbon precursor and synthetized through green‐chemistry techniques with a novel aqueous‐based electrospinning process whilst co‐spinning lignosulfonate with sacrificial gelatin. We consider hard carbon as a composite of sp^2^ and sp^3^ hybridized carbon, the proportion of these two types of carbon hybridization is key to an optimized material for sodium‐ion batteries. We provide important insights into process optimization at nanoscale for electrochemical performance whilst ensuring sustainability of hard carbon via the usage of lignosulfonate. While no synthetic binders or conductive additives were utilized in the fabrication of the electrodes. Additionally, this approach expands the potential applications and value of lignosulfonate, which is currently an undervalorized raw material.

## 2 Results and Discussion

To obtain electrospun lignosulfonate/gelatin fibers, first, a study of the electrospinning of natural polymers dissolved in water was performed, (see Table S1). Gelatin is blended with lignosulfonate, with no requirement for additional crosslinkers to maintain structural integrity at room temperature. As the gelatin is sacrificial, it is important to assess structural damage of resulting fibers upon its rapid removal during stabilization. Therefore, stabilization and carbonization conditions were optimized to produce carbon nanofibers. Polydiallyldimethylammonium chloride (PDADMAC) was added at varying levels 0.25%–0.5%–0.75%, acting as an interface stabilizer to allow ease of spinning of the aqueous solution. Two protocols were investigated: stabilization with an initial isotherm of 150°C and direct carbonization.

To assess if the temperature of the initial isotherm during stabilization had an impact on the stability of the fibers compared to the total absence of a stabilization step, different stabilization protocols were applied to the fibers (Table S1). The interface stabilizer was fixed at 0.25% PDADMAC as this showed the best structural integrity in the earlier electrospinning experiment. Figure S2 shows scanning electron microscope (SEM) X‐ray diffraction images of fibers synthesized from 25% gelatin, 15% lignosulonate, and 0.25% PDADMAC taken using each stabilization temperatures and subsequent carbonization. Good fiber stability and homogeneity can be observed in all the different stabilization protocols, see supplementary data Figures S3–S10.

Stabilization significantly impacts fiber diameter by controlling thermal shrinkage, cross‐linking, and phase separation. In a system with 20%–25% gelatin as a sacrificial material and 15% lignosulfonate as a hard carbon precursor, stabilization dictates the degree of structural integrity of the fibers before pyrolysis. Lignosulfonate undergoes oxidation and cross‐linking, forming a rigid network that prevents excessive fiber shrinkage. Analysis of the fibers show that stabilization at a first isotherm of 150°C (G150) yields fibers with a larger diameter and higher standard deviation than the lower temperature isotherm at 100°C (G100 – Table 1). However, direct carbonization (G) gives the highest standard deviation in fiber diameter and largest fiber diameter using the composition of 20% gelatin and 15% lignosulfonate. The most homogeneous, smaller in diameter and non‐beaded fibers are a result of the composition of 25% gelatin, 15% lignosulfonate and 0.25% PDADMAC with an initial isotherm of 40°C and 100°C (G40 and G100) during stabilization. This means that the lower temperature helps maintain fiber homogeneity and smaller fiber diameters. When no stabilization step is present (G), the fiber diameter's standard deviation and fiber diameter dramatically increase. This can be attributed to the fact that the quick input of thermal energy generates more void and hollow parts whereas, when stabilization is applied, the carbon precursor has more time to reorganize and more compressed structures are formed, portraying smaller fiber diameter.

**TABLE 1:** Fiber diameter and Standard Deviation (STD) of the most stable fibers after stabilization and carbonization.

Sample			Fiber diameter (nm)
15% Lignosulfonate 0.25% PDADMAC	25% Gelatin 25% Gelatin 25% Gelatin 25% Gelatin 20% Gelatin 20% Gelatin 20% Gelatin 20% Gelatin	G40	190 ± 105
		G100	200 ± 100
		G150	240 ± 90
		G	900 ± 200
		G40	260 ± 110
		G100	286 ± 90
		G150	300 ± 100
		G	1200 ± 300

The results of the fiber diameter state that 25% gelatin had a smaller fiber diameter when compared to the 20% gelatin fibers undergoing the same thermal treatments, smaller diameter is thought to improve sodium‐ion dynamics and storage mechanisms. For battery applications, smaller fiber diameter increases the active surface in contact with the electrolyte and improves mechanical stability and stress accommodation as they reduce the risk of degradation over cycling [24]. As a result of this, 25% gelatin, 15% lignosulfonate and 0.25% PDADMAC were selected as the optimal composition, and future tests were carried out using that composition as baseline. X‐ray diffraction (XRD), Raman, and X‐ray photoelectron spectroscopy (XPS) were performed on the optimized composition of gelatin and lignosulfonate fibers to confirm the obtention of hard carbon via different stabilization and carbonization protocols.

**FIGURE 1:**
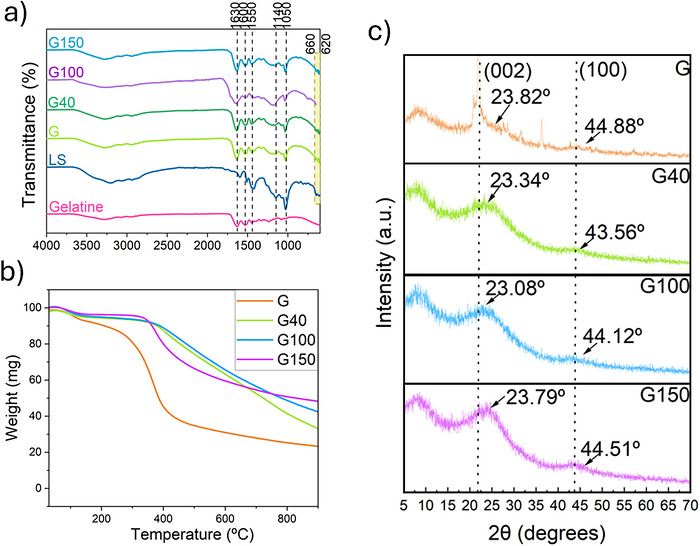
(a) FT‐IR spectrum of lignosulfonate and gelatin, as spun fibers and stabilized samples; (b) TGA analysis of G, G40, G100, and G150; (c) XRD of carbonized G, G40, G100, and G150.

FT‐IR spectroscopy was performed to verify the presence of gelatin and lignosulfonate in the samples before and after stabilization (Figure 1a). The characteristic fingerprint region of Lignosulfonate (LS) is located within the range of 1900 to 600 cm^−1^ [25]. Peaks at 1600 and 1500 cm^−1^ refer to C═C skeletal vibrations. The peaks present at 1470, and 1460 cm^−1^ show the C─H deformation within the aromatic ring, a common structure found at technical lignins. The peaks at 1260 and 1140 cm^−1^ stand for the C═O stretch and C─H in‐plane deformation, respectively. Complex vibrations associated with C─O, C─C stretching, and C─OH bending is represented through the peak at 1050 cm^−1^. The peaks from 660 to 620 cm^−1^ refer to the sulfonic groups in lignosulfonate, CS─O stretching vibrations. Amide I is found at 1630 cm^−1^, corresponding to C─O and C─N stretching. The peak at 1550 cm^−1^ shows the NH bending band of amide II. The peak between 1240 and 1250 cm^−1^ is the amide III band, standing for C─N stretching and N─H bending.

TGA analysis was performed to determine the carbon yield of the carbon precursor. The thermal properties of the stabilized fibers are shown in Figure 1b. Samples G and G150 experience weight loss that can be found between 380°C and 500°C, which is attributed to the degradation of C─C bonds [26]. This is the most severe in sample G, and it can be attributed to the fact that no previous thermal stabilization was applied to this sample which promotes extensive precursor decomposition in comparison with the stabilized samples. Thermal cross‐linking of the carbon precursor is more effective in samples that undergo stabilization as the polymer chains have more time to cross‐link and so the carbon yield is higher than in sample G, in which no stabilization step was performed. The carbon yield is 23.28%, 33.07%, 42.36%, and 48.19% for G, G40, G100 and G150, respectively.

XRD, Raman, and XPS were performed on the optimized composition of gelatin and lignosulfonate fibers to confirm the obtention of hard carbon via different stabilization and carbonization protocols. The wide‐angle XRD spectra for the carbonized samples are shown in Figure 1c. XRD is the preferred technique to characterize the crystalline structure and the lattice parameters of the material under study. The peaks of interest in hard carbon are present at 22° and 43°, corresponding to the (002) and (100) crystal planes of turbostratic and disordered 2H graphite, respectively [4]. All the samples show wide peaks in the areas of interest, 22° and 43°. However, sample G presents some narrow peaks, these narrow peaks can be found around 21° and 36°, which correspond to sodium and SiO_2_ impurities found in sample G due to its direct carbonization. The presence of these impurities is confirmed by the results obtained with the XPS analysis of the samples. Interplanar distance for the peak at 22° was calculated for all samples applying Equation 1: Bragg's Law equation. Plane (002), shown with the peak at 22°, was selected as it relates to the crystal planes of graphite‐like carbon, resulting in the main interlayer's reflection. On the other hand, plane (100), at 43°, loses layer‐to‐layer crystalline coherency as it represents the more amorphous regions of the hard carbon [4].

(1) $$n\lambda =2dsin\left(\theta \right)$$

Consider *n* = 1, referring to the order of reflection; λ = wavelength, in this case, 0.154 nm; *d* = interplanar distance, θ = angle between the planes and the incident rays. To calculate the interplanar distance the peak at 22° is used as it is the main interlayer reflection. Based on 2θ values of 23.82°, 23.34°, 23.08°, and 23.79° interplanar distances of 0.373, 0.380, 0.385, and 0.374 nm were calculated for the G, G40, G100, and G150 samples respectively.

Based on the principle that, hard carbon is composed of a combination of pseudo‐graphitic microdomains, sp^2^ bonded carbon, and amorphous regions, sp^3^ bonded carbon [4], the results Raman and XPS are analysed to try and understand the proportion of those hybridizations in each sample. Hence, relating structure with performance in a hierarchical way.

**FIGURE 2:**
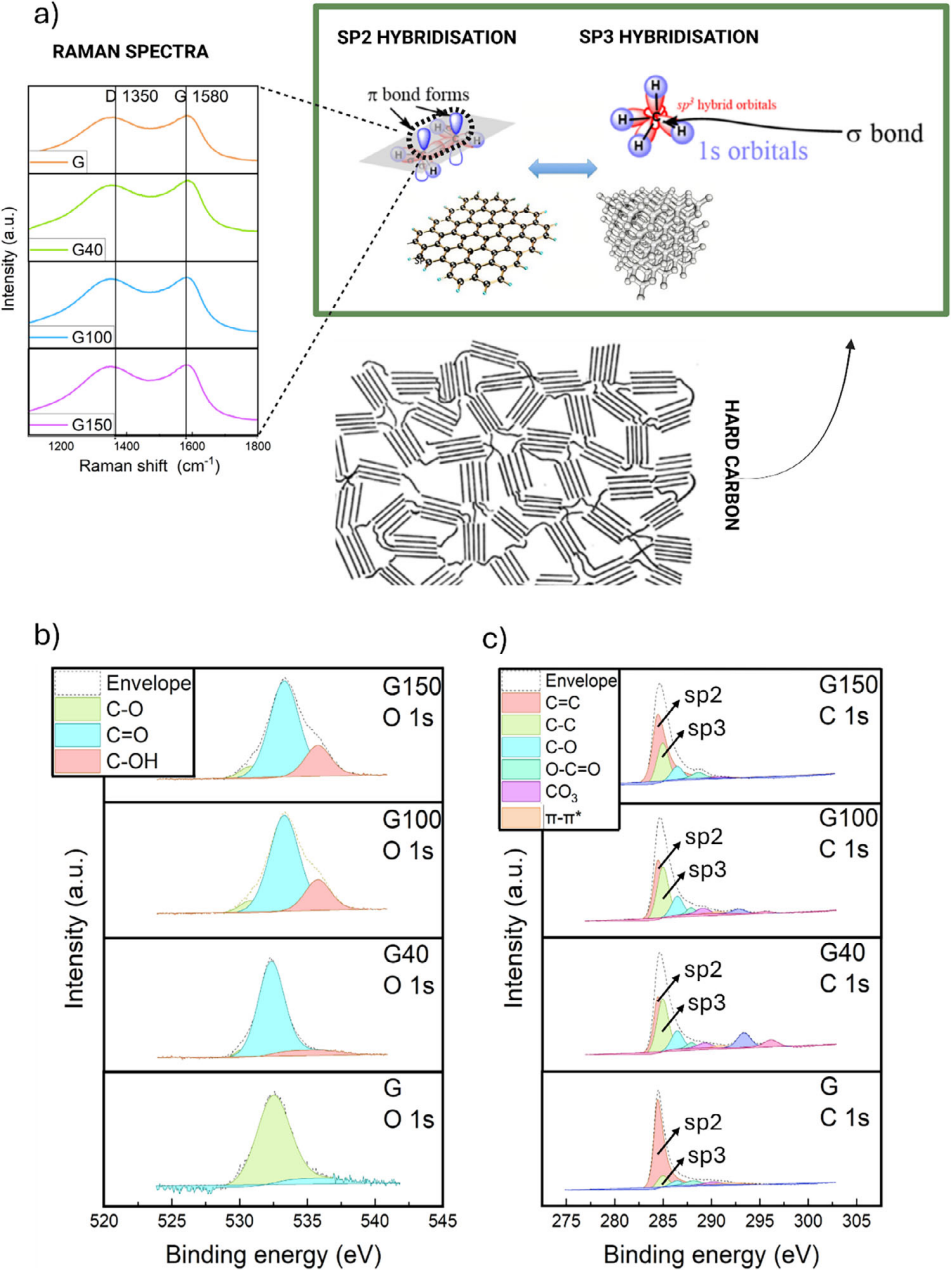
(a) Raman of carbonized G, G40, G100, and G150. XPS of G150, G100, G40, and G, centered for carbon and oxygen. (b) High‐resolution XPS for O 1s; (c) High‐resolution XPS for C 1s.

Raman spectra were taken for the carbonized samples to confirm that the presences of hard carbon after carbonization (Figure 2a). Raman spectroscopy was performed to analyze the degree of graphitization in the samples. An incident monochromatic light of wavelength 512 nm was chosen as it resonates with *π*‐satellite electrons, present only in the sp^2^ carbons of the graphene layers, whereas it is unaffected by sp^3^ hybridized carbon [27]. Hence, it is a useful technique to determine the degree of defects and disorder present in sp^2^ carbons, see Figure 2 [28]. In all analyzed samples, two main peaks are present at 1350 and 1580 cm^−1^, corresponding to the characteristic D and G bands in hard carbon. The D band refers to the breathing modes of sp^2^ carbon within rings and defects, whereas the G band is associated with bond stretching of all pairs of sp^2^ carbons and graphitic order. When studying the intensity of peaks, the I_D_/I_G_ ratio can be calculated to define the degree of defects and disorder within the graphitic regions of the samples [29], the degree of the disorder decreases with the ratio. The results were obtained by fitting the peaks of the D and G bands and performing a simple calculation. The I_D_/I_G_ intensity ratio calculated is 0.39, 0.82, 0.88, and 0.82 for sample G, G40, G100, and G150, respectively. This demonstrates that samples G40, G100 and G150 present small, disordered sp^2^ domains due to their high ratio. On the other side, sample G is more characterized by larger, more ordered graphitic regions.

High‐resolution XPS was performed to deeply analyze the chemical state of individual atoms, specifically carbon and oxygen, see Figure 2b,c. The C 1s spectra are fitted by six peaks located at around 284, 285, 286, 288, 290, and 292 eV corresponding to the C─C/C═C, C─O, C═O, O─C═O, CO_3_, and *π*–*π* satellite, respectively. The high‐resolution spectra of O 1s for all samples show two peaks located around 532.9 and 535.8 eV that refer to Csp2═O and Csp3─OH [5]. The lignosulfonate precursor contains impurities, which infer water solubility [30].

The C 1s spectra can differentiate between the peaks corresponding to sp^2^ and sp^3^ carbon [5]. Carbon bonded in C═C, sp^2^ carbon, shows a peak with a binding energy of 284.4–284.6 eV. Carbon bonded in C─C, sp^3^ carbon, presents a peak with a binding energy of 285.0–285.2 eV. Table 2 shows the area of the XPS peak of sp^2^ and sp^3^ carbon after considering the signal contributions introduced by the oxygen bonds, as well as the relative percentage of sp^2^ and sp^3^ carbon present in each sample. The area of the XPS peaks is used to calculate the sp^3^/sp^2^ ratio that determines the proportion of sp^2^ and sp^3^ carbon present in the sample. The sp^3^/sp^2^ ratio calculated is 0.36, 0.82, 0.71, and 0.39 for samples G, G40, G100, and G150, respectively.

**TABLE 2:** XPS analysis of G, G40, G100, and G150.

Sample	Carbon	Type of bond	Area of the XPS peak	Relative %	sp^3^/sp^2^ Ratio
G	sp^2^	C═C	41130	54.3	0.36
	sp^3^	C─C	15151	20.0	
G40	sp^2^	C═C	32758	37.9	0.82
	sp^3^	C─C	27076	31.4	
G100	sp^2^	C═C	26152	37.2	0.71
	sp^3^	C─C	18601	26.4	
G150	sp^2^	C═C	32415	48.8	0.39
	sp^3^	C─C	12838	19.3	

We propose that the initial isotherm during stabilization plays a crucial role in governing the structural evolution of the resulting carbon material. We hypothesize that when stabilization occurs at a lower initial isotherm (e.g., G40 and G100), the energy supplied may be insufficient to drive extensive decomposition of oxygen‐containing groups in lignosulfonate. This leads to slow and incomplete oxidation, resulting in a more disordered cross‐linked network. Although prolonged stabilization theoretically allows for increased cross‐linking, it also promotes structural heterogeneity, potentially hindering the formation of pseudographitic sp^2^ hybridized carbon during carbonization. This aligns with our observations of higher *I_D_/I_G_* ratios (0.82 and 0.88) and elevated sp^3^/sp^2^ ratios (0.82 and 0.71) in G40 and G100, indicating a greater fraction of disordered carbon and sp^3^ bonding. The increased disorder may contribute to a higher density of defect sites, which could enhance specific capacity.

Conversely, when stabilization is performed at a higher initial isotherm (e.g., G150), we believe that the rapid influx of thermal energy facilitates a more efficient and selective bond rearrangement. This process likely promotes the formation of shorter but more structurally organized cross‐linked regions, which may be characterized as pseudographitic microdomains. The increased energy input could accelerate cross‐linking reactions and promote early aromatization, leading to a higher proportion of sp^2^ carbon, as reflected in the lower sp^3^/sp^2^ ratio (0.39). This suggests that the transition from sp^3^ tetrahedral bonding to sp^2^ planar configurations occurs earlier, driven by the availability of sufficient thermal energy to facilitate delocalized *π*‐electron formation. Ultimately, while extended stabilization times (as seen in G40 and G100) might theoretically enhance cross‐linking opportunities, we hypothesize that the degree of structural organization within the cross‐links is a more critical factor in determining the final carbon structure.

XPS analysis focused on C 1s is utilized to determine the proportion of redox‐active groups on the surface of the samples, as the main oxygenated functional groups have overlapping binding energy in the O 1s, Table 3.

**TABLE 3:** XPS analysis of G, G40, G100 and G150.

Sample	Redox group	Type of bond	Relative %	Total of redox active groups
G	Carboxyl	O─C═O	7.9	12.86%
	Hydroxyl	C─O	3.8	
G40	Carboxyl	O─C═O	10.8	13.18%
	Hydroxyl	C─O	2.7	
G100	Carboxyl	O─C═O	9.8	21.64%
	Hydroxyl	C─O	2.6	
G150	Carboxyl	O─C═O	6.5	16.23%
	Hydroxyl	C─O	3.9	

We propose that in sodium‐ion batteries, redox sites (carboxyl and hydroxyl groups as shown in Table 3) provide extra sites for sodium‐ion storage, as with the reduction of these sites during discharge they can reversibly bind to the sodium‐ion. Hence, enhancing the specific capacity of the material as these groups can act as secondary active sites [31]. The area under the peaks of the C 1s spectra of the samples G, G40, G100, and G150 is used to calculate the relative percentages shown in Table 3. The formation of carboxyl and hydroxyl groups depends on oxidation reactions, which have activation energy thresholds [32]. Among the samples studied, G100 and G150 are the ones that present the highest percentage of redox functional groups with 21.64% and 16.23%, respectively. Showing that those conditions may be optimal for oxidation reactions to introduce, organize or stabilize oxygen‐containing functional groups among lignosulfonates.

**FIGURE 3:**
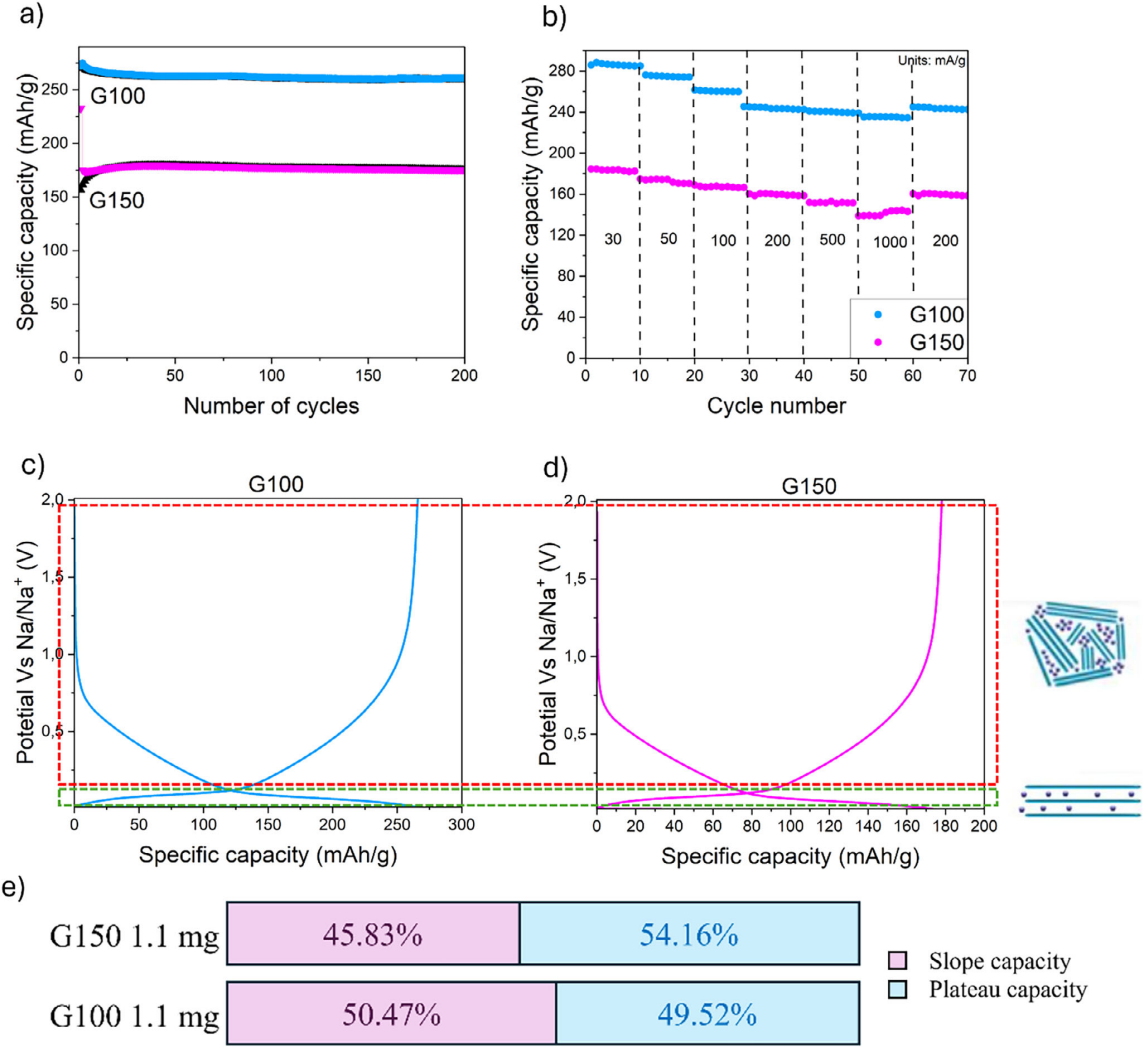
Electrochemical testing of G100 and G150 as SIB anode materials, 2.4 and 2.1 mg/cm^2^ mass loading, respectively. (a) cyclic performance of G100 and G150 samples at 100 mA g^−1^; and (b) rate capabilities of G100 and G150 at different current densities. (c) GCD curves of the 10th cycle of G100; (d) and G150; (e) Graphic showing the percentages of slope and plateau capacity that define G100 and G150.

For the following electrochemical testing, samples G100 and G150 are selected. Long‐term cycling was performed at 100 mA/g as shown in Figure 3a,b, to assess the electrochemical performance of G150 and G100 over 200 cycles. G150 electrode exhibits a stable capacity of 173.5 mAh/g with 99.8% Coulombic efficiency (CE) over 200 cycles when having a mass loading of 2.4 mg/cm^2^. The CE of the 1st and 2nd cycles is 98.8% and 99.1%, respectively. G100 electrode exhibits a stable capacity of 264.3 mAh/g with 99.9% CE over 200 cycles when having a mass loading of 2.1 mg/cm^2^. The CE of the 1st and 2nd cycles is 99.2% and 99.4%, respectively. A mass loading study was performed and can be found in Figures S11–S13. Table 4 compares performance levels with state‐of‐the‐art.

**TABLE 4:** Comparison of bio‐derived carbons for sodium‐ion batteries.

System	Precursor	Specific capacity	Coulombic efficiency
G150	Lignosulfonate	173.5 mAh/g after 200 cycles	CE 1st/2nd cycle: 98.8% / 99.1%; average 99.8% over 200 cycles.
G100	Lignosulfonate	264.3 mAh/g after 200 cycles	CE 1st/2nd cycle: 99.2% / 99.4%; average 99.9% over 200 cycles.
Lignin‐derived HC [33]	Lignin from pulping	307–336 mAh/g at low current; stable over 250 cycles	Initial CE around 80%–85%; CE approaches 100% in subsequent cycles
Lignin‐derived partially graphitic HC [34]	Larch wood lignin	Initial discharge 350 mAh/g; improved capacity retention vs cellulose HC, with 84% retention after 100 cycles at 0.5 C	Initial CE 74%; CE near 100% during subsequent cycling
Lignin molecular‐sieving HC [35]	Pine lignin fractions	300 mAh/g‐level reversible capacity with good stability (100–200 cycles)	Initial CE typically <85%; stabilized CE 99% after formation cycles
Silicon‐doped biomass HC [36]	Distillers’ grains	280.6 mAh/g with 102.6% capacity retention after 100 cycles	Initial CE markedly improved vs. undoped HC, reaching 70%–80%, with later CE 100%
Lignin/cellulose‐derived HCs [37]	Lignin cellulose	Typical reversible capacities 200–300 mAh/g after 100–200 cycles	Initial CE often 60%–80%; CE approaches 100% after several cycles.
Honeycomb‐like HC from pine pollen [38]	Pine pollen	203 mAh/g after 200 cycles, good capacity retention	CE quickly stabilizes near 100% after initial cycles
Biomass‐derived porous HC [39]	General biomass	181 mAh/g after 220 cycles	CE 100% after initial conditioning cycles

The rate capability of each sample was performed at 30, 50, 100, 200, 500, and 1000 mA g^−1^ with the results presented in Figure 3b. G100 presented 285.9, 274.8, 260.2, 243.4, 240.5, 235.6, and 243.4 mA g^−1^ respectively. While G150 presented 183.7, 174.4, 165.1, 159.7, 151.0, 142.2, and 159.7 mA g^−1^ respectively, at each current density. When the current density reverted to 200 mA g^−1^ after 60 cycles, the capacity returned to 243.4 and 159.7 mAh g^−1^ for G100 and G150, respectively. The low variation of specific capacity during different charge/discharge rates in G100 can be explained due to its small fiber diameter (200 ± 100 nm).

By analyzing the voltage profiles, we determine the percentage of slope and plateau capacity for the samples (Figure 3c,d). G100 exhibits 50.47% slope and 49.52% plateau capacity, while G150 shows 45.83% slope and 54.16% plateau capacity (Figure 3e). Despite the significant difference in sp^3^/sp^2^ ratios (0.71 for G100 and 0.39 for G150), the slope‐plateau percentages remain close due to structural heterogeneity and the gradual transition between disordered and graphitic domains. While G100 has a higher sp^3^ content, it does not mean that it is entirely amorphous, nor does G150 consist purely of graphitic domains. Instead, both materials contain a mix of sp^3^ and sp^2^ regions, creating a continuous transition in electrochemical behavior rather than a strict separation between slope‐ and plateau‐dominant capacities.

**FIGURE 4:**
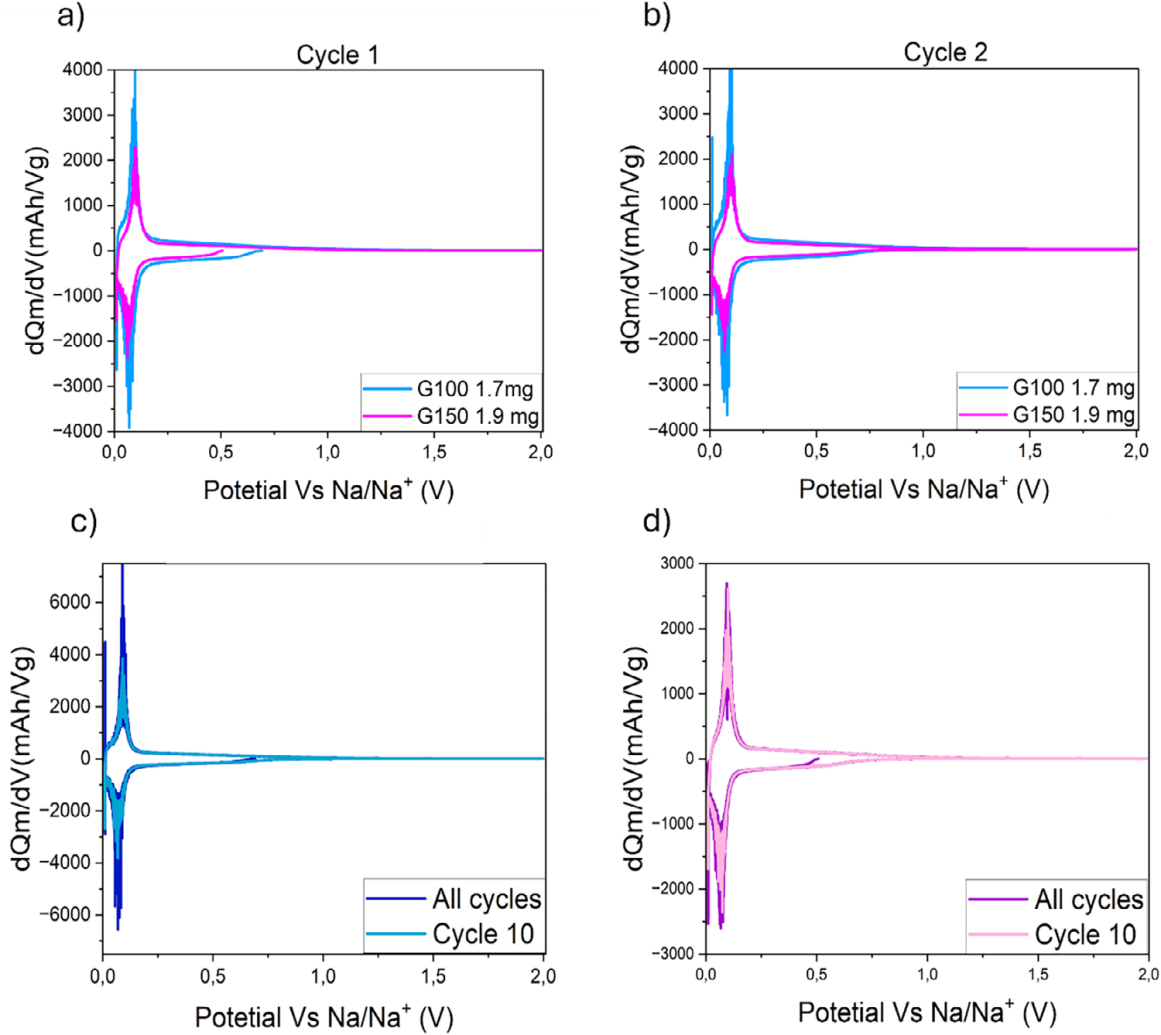
Electrochemical testing of G100 and G150 as SIB anode materials, 2.4 and 2.1 mg/cm^2^ mass loading, respectively. The differential capacity plot of G100 and G150 electrodes (electrodes were cycled at 100 mA g^−1^ in the potential range of 0.01−2.0 V): (a) Cycle 1; (b) Cycle 2; (c) G100 all cycles and Cycle 10, (d) G150 all cycles and Cycle 10.

In Figure 4a,b, the differential capacity plots are represented, the first and the second cycles show the formation of the solid‐electrolyte interface (SEI) which corresponds to the decomposition of the electrolyte. In the first sodiation (discharge), a broad peak between 0.20–0.75 V in the differential capacity plot indicates electrolyte decomposition and formation of the SEI layer, leading to an initial coulombic efficiency (ICE) of 98.3% and 99.7% for G150 and G100, respectively, considering a mass loading of 2.4 and 2.1 mg/cm^2^, respectively. The dQm/dV curves for the 10^th^ cycle and all cycles together can be seen in Figure 4c,d. The 10^th^ cycle, which shows a corresponding broad peak between 0.2 and 0.75 V in both samples, is related to the gradual voltage drop and a sharper peak <0.1 V related to the plateau region. A stable SEI layer was formed in all the batteries due to having stable capacity plots maintained over time [40], see Figure S11.

We propose that structural evolution during stabilization significantly influences the electrochemical performance of the resulting carbon materials. Our data suggests that stabilization conditions impact the balance between sp^3^ and sp^2^ hybridized carbon domains, affecting sodium‐ion storage behavior. To explain the difference in performance of the G100 and G150 hard carbon fiber anodes, it is useful to compare the distinct structural features that may correlate with the capacity difference. Three key disparities are observed, with the G100 fibers exhibiting a: (i) higher sp^3^/sp^2^ and *I_D_/I_G_* ratios; (ii) larger interlayer spacing; and (iii) thinner fiber diameter. These structural differences likely work in combination to influence sodium storage mechanisms and ultimately the performance of each anode. The higher sp^3^/sp^2^ and *I_D_/I_G_* ratio in G100 signifies greater structural disorder, which can introduce a higher concentration of defect sites and pore surfaces for sodium‐ion storage [41]. Furthermore, G100's expanded interlayer spacing (0.385 nm) significantly exceeds the ∼0.37 nm minimum required for efficient Na storage into carbon layers [42]. The thinner fiber diameter with larger concentrations of oxygen‐containing functional groups in G100 are likely to contribute to its higher capacity by shortening sodium‐ion diffusion paths and increasing the effective contact area with electrolyte [4, 43].

**FIGURE 5:**
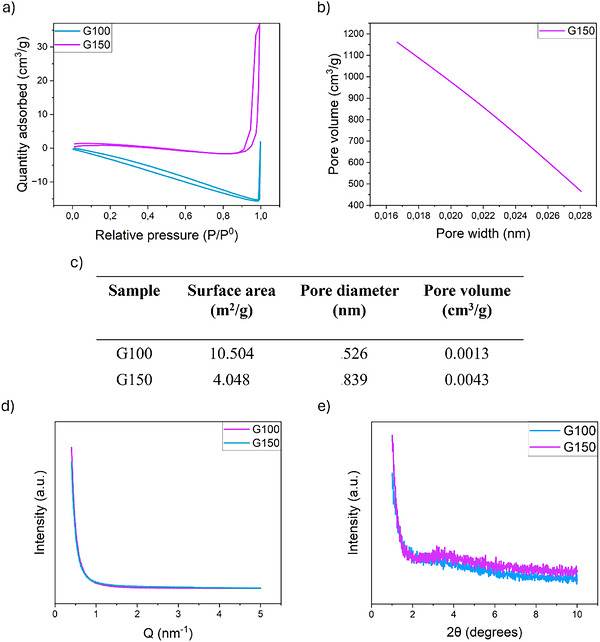
(a) N_2_ adsorption–desorption isotherms, (b) Pore diameter distribution, (c) BET analysis of G100 and G150. Powder X‐ray analysis of G100 and G150: (d) SAXS and (e) Low angle XRD.

Additionally, to better assess the causes of different behaviors of both samples during the electrochemical testing for sodium‐ion batteries, SAXS and BET analyses were conducted. Figure 5 compares the textural and structural characteristics of samples G100 and G150. The N_2_ adsorption–desorption isotherms (Figure 5a) reveal a higher adsorbed volume and more pronounced hysteresis for G150, indicating the presence of larger mesopores compared to G100. This is consistent with BET results (Figure 5c), where G150 shows a slightly lower surface area (4.0 m^2^/g) but a higher pore volume (0.0043 cm^3^/g) and larger average pore diameter (0.839 nm) than G100 (10.5 m^2^/g, 0.0013 cm^3^/g, 0.526 nm). The pore size distribution (Figure 5b) further confirms this shift toward broader pores in G150. SAXS and low‐angle XRD patterns (Figure 5d,e) show similar scattering profiles for both samples, suggesting that while the mesoporous texture evolves with synthesis conditions, the overall nanostructural order remains comparable between G100 and G150.

To rationalize the differences in sodium storage between the G100 and G150 materials, a series of density functional theory (DFT)‐based quantum‐chemical calculations were performed on coronene‐derived carbon models designed to reflect the hybridization ratios measured via XPS. All calculations were carried out using Gaussian 16c [44] at the B3LYP/6‐31+G(d,p) [45, 46] level of theory, including counterpoise correction to remove basis‐set superposition error (BSSE) [47]. Sodium was modelled as Na^+^, consistent with the electrolyte conditions.

**FIGURE 6:**
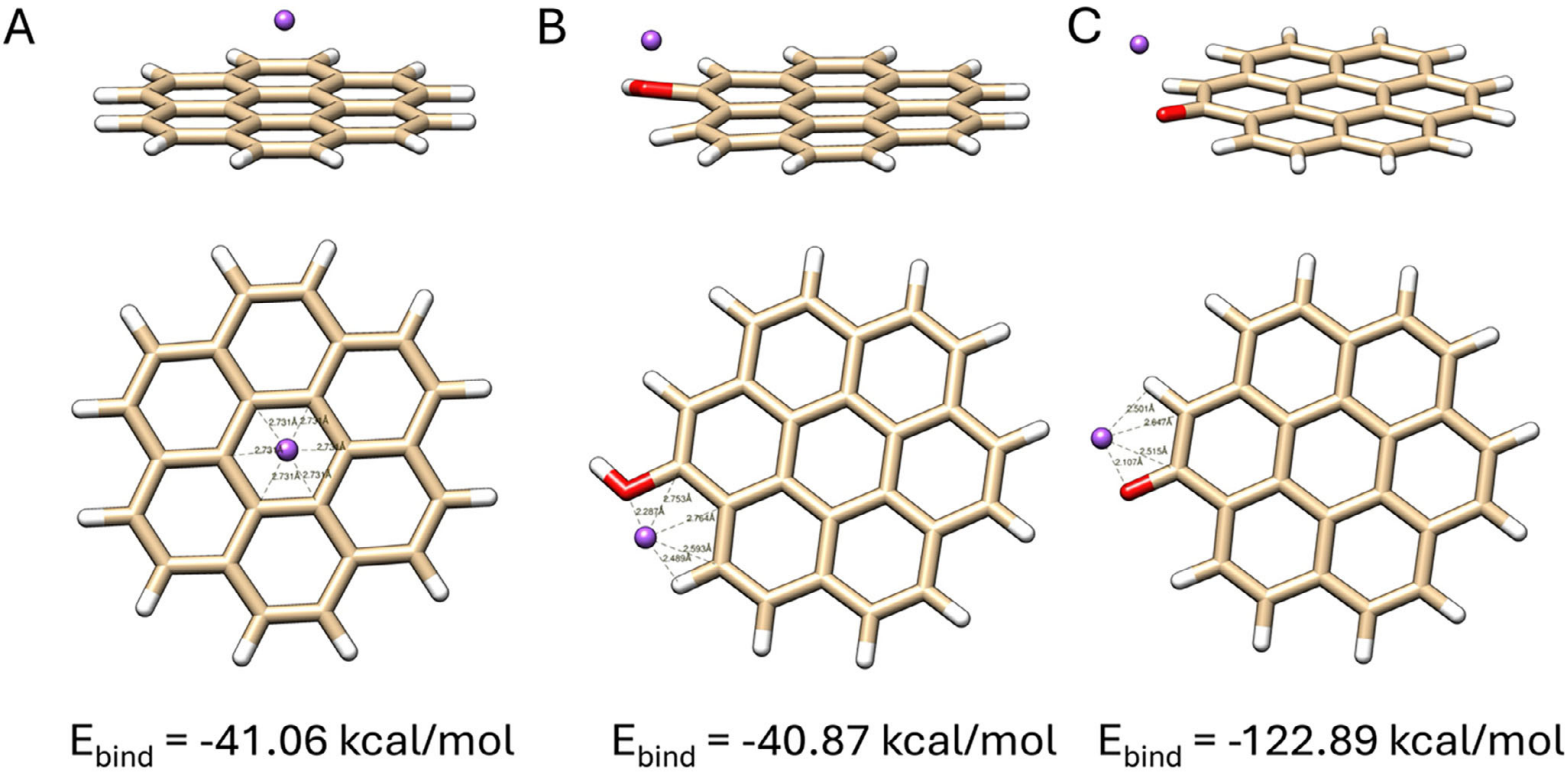
DFT‐optimized geometries and Na^+^ binding energies for three representative carbon surface models. (a) Pristine coronene (pure sp^2^ domain) showing classical cation‐*π* stabilization of Na^+^ located above the aromatic ring center (E_bind_ = −41.06 kcal mol^−^^1^). (b) Single ─OH edge‐defect coronene (mixed sp^2^/sp^3^ environment) where Na^+^ coordinates simultaneously to the *π*‐system and the defect oxygen site, yielding a comparable binding energy (E_bind_ = −40.87 kcal mol^−^^1^). (c) Vacancy‐type defect without ─OH (sp^3^‐rich cavity) exhibiting substantially enhanced Na^+^ binding (E_bind_ = −122.89 kcal mol^−^^1^), consistent with strong electrostatic stabilization within the defect pocket. All geometries were optimized at the B3LYP/6‐31+G(d,p) level of theory, and binding energies were computed using counterpoise‐corrected single‐point calculations on the optimized complexes. Distances in Å highlight the Na^+^ coordination environment for each model.

Three structural models were selected (see Section S1 and Table S3):

1. Pristine Coronene (sp^2^‐rich graphitic domain) (Figure 6a). This system represents extended aromatic surfaces characteristic of G150, where the XPS data indicate a dominant sp^2^ contribution. Na^+^ was placed above the central hollow site of the coronene ring. After optimization, Na^+^ remained weakly bound via cation–*π* interactions, yielding a BSSE‐corrected binding energy of −41.06 kcal/mol.
2. OH‐Functionalized Coronene (mild sp^3^/oxygenated edge) (Figure 6b). This structure introduces a single OH group at an edge carbon to mimic localized oxidation or partial sp^3^ character. In this orientation, Na^+^ interacts weakly with the surface, and the binding energy (−40.87 kcal/mol) is nearly identical to that of pristine coronene. This indicates that the presence of a single OH group does not significantly enhance Na^+^ affinity under the sampled conditions.
3. Vacancy‐Type Defective Coronene (sp^3^‐rich defective cavity) (Figure 6c). To model the highly defective, sp^3^‐enriched microstructure of G100 (sp^3^/sp^2^ = 0.71), a vacancy‐type defect was introduced by removing an edge carbon and protonating the adjacent site. Na^+^ binds within the resulting asymmetric cavity through multi‐point interactions with distorted carbon frameworks. This model exhibits very strong Na^+^ stabilization, with a BSSE‐corrected binding energy of −122.89 kcal/mol.

Thus, the enhanced electrochemical performance of G100 arises from its greater density of defect sites capable of forming deep Na^+^ adsorption pockets. This provides a strong mechanistic explanation linking carbon hybridization, defect chemistry, and sodium‐storage behavior. Similar DFT frameworks have recently demonstrated how cation‐*π* interactions and outer‐sphere ionic environments modulate charge transport pathways in molecular electronic materials [48].

While several models have been proposed to describe sodium storage in hard carbon, there remains ongoing debate regarding the dominant mechanisms and the specific roles of defects, interlayer spacing, and porosity. These findings align most closely with the model proposed by Cao et al., where sodium‐ions are adsorbed on the surface, defects, and pores in the slope region, and sodium ions are intercalated into carbon layers in the plateau region [49].

## 3 Conclusion

This study presents an innovative green chemistry synthesis route for nanostructured lignosulfonate, which is soluble in water and presents surfactant‐like properties. Upon carbonization these fibers are utilized in energy storage applications. We show that processing conditions are key to sp^2^ / sp^3^ hybridized carbon optimization, which critically influences the electrochemical properties of the hard carbon. We demonstrate the potential application of these fibers in sodium‐ion batteries. Our findings appear to align most closely with the model proposed by Cao et al., where sodium‐ions are adsorbed on the surface, defects, and pores in the slope region, and sodium ions are intercalated into carbon layers in the plateau region. In this context, the more pronounced slope region observed for G100 may be linked to its higher sp^3^/sp^2^ ratio, which reflects greater structural disorder and a higher density of surface sites, defects, and edge functionalities that could serve as adsorption sites. Additionally, G150's greater proportion of sp2 carbons allows for a higher degree of intercalation into the turbostratic graphitic domains and hence a more pronounced plateau region in the voltage profile. Although our data are consistent with Cao's model, distinguishing between competing mechanisms remains challenging.

## Conflicts of Interest

The authors declare no conflicts of interest.

## Supporting information




**Supporting File**: adma72508‐sup‐0001‐SuppMat.docx.

## Data Availability

The data that support the findings of this study are available from the corresponding author upon reasonable request.
