# Supplementary material for: A Mechanistic Study of Bio‐Based Nanotemplated Carbon Nanofibers Derived From Water Processable Lignin Blends for Sustainable Energy Storage Applications
Source: Adv Mater. 2026 Feb 11;38(39):e72508. doi: 10.1002/adma.72508 (PMC13361255; doi:10.1002/adma.72508)
Supplement: Supplementary file 1 — Supporting File: adma72508‐sup‐0001‐SuppMat.docx. [file ADMA-38-e72508-s001.docx]

**SUPPLEMENTARY INFORMATION**

**A Mechanistic Study of bio-based nanotemplated carbon nanofibers derived from water processable lignin blends for sustainable energy storage applications**

Judith Miralda Jalle^1,4^, Jamal El Haskouri^2^, Anne Beaucamp^1,4^, Tadhg Kennedy^3,4^, Mario Culebras^2^, Maurice N. Collins^1,4^

^1^ Stokes Laboratories, School of Engineering, University of Limerick, Limerick, Ireland

^2^ Institute of Material Science, Universitat de València, Valencia, Spain

^3^ Department of Chemical Sciences, University of Limerick, Limerick, Ireland

^4^ Bernal Institute, University of Limerick, Limerick, Ireland

**
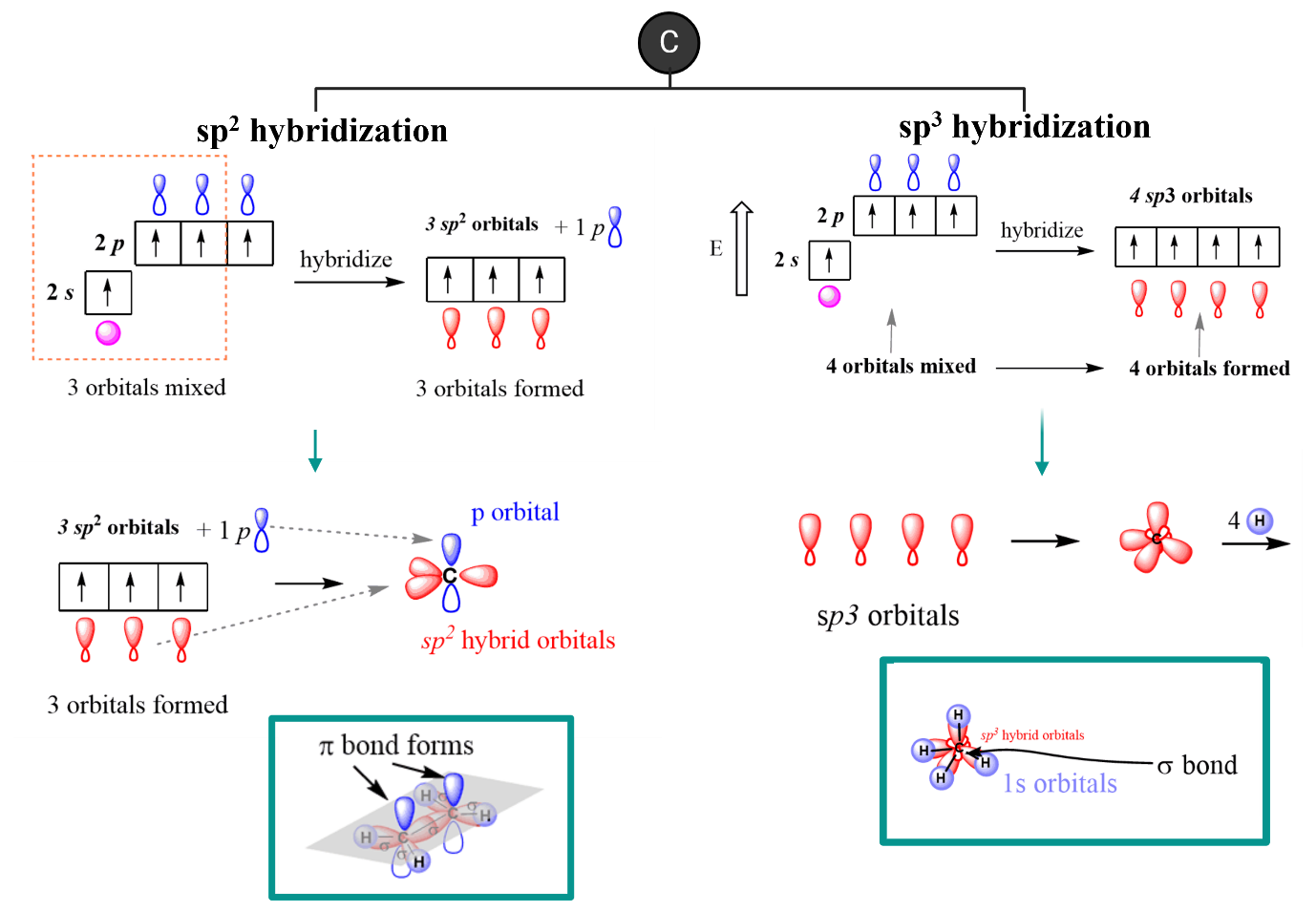
**

**Figure S1. Carbon hybridization diagram**

**EXPERIMENTAL SECTION**

**Materials**

Alginic acid sodium salt (MW = 10,000–600,000 g/mol) was purchased from AppliChem PanReac (Lennox Ireland). Gelatin from porcine skin powder, gel strength ~300 g Bloom, Type A and Poly (diallyl dimethylammonium chloride) (PDADMAC) solution ($M$*_W_*=200,000-350,000; 20 wt. % in H_2_O) were purchased from Sigma-Aldrich (Ireland). Ammonium lignosulfonate was purchased from Lignotech Florida.

**Optimization of the preparation of Lignosulfonate/gelatin fibers**

Different compositions of gelatin and alginate in water and 2% acetic acid in water were electrospun. The best composition was selected, 15 wt% of lignosulfonate was added to 20 and 25 wt% of gelatin solutions with varying percentages of PDADMAC (0, 0.25, 0.5, and 0.75 wt%). The lignosulfonate powder was weighed and then added into vials and the PDADMAC was subsequently added. This solution was kept under continuous stirring overnight at 40 °C. The composition based on 25 wt% of gelatin, 15 wt% of lignosulfonate, and 0.25% of PDADMAC dissolved in water is selected to prepare the carbon nanofibers (CNFs).

**Preparation of the CNFs**

The preparation of the CNFs consists of 3 different steps, see Figure S2. Inovenso NE300 was used to produce the CNFs. The samples were 25 wt% of gelatin, 15 wt% of lignosulfonate, and 0.25% of PDADMAC dissolved in water and produced at a voltage of 30 KV and a collector-spinneret distance of 150 mm, electrospinning for 30 minutes**.**

**
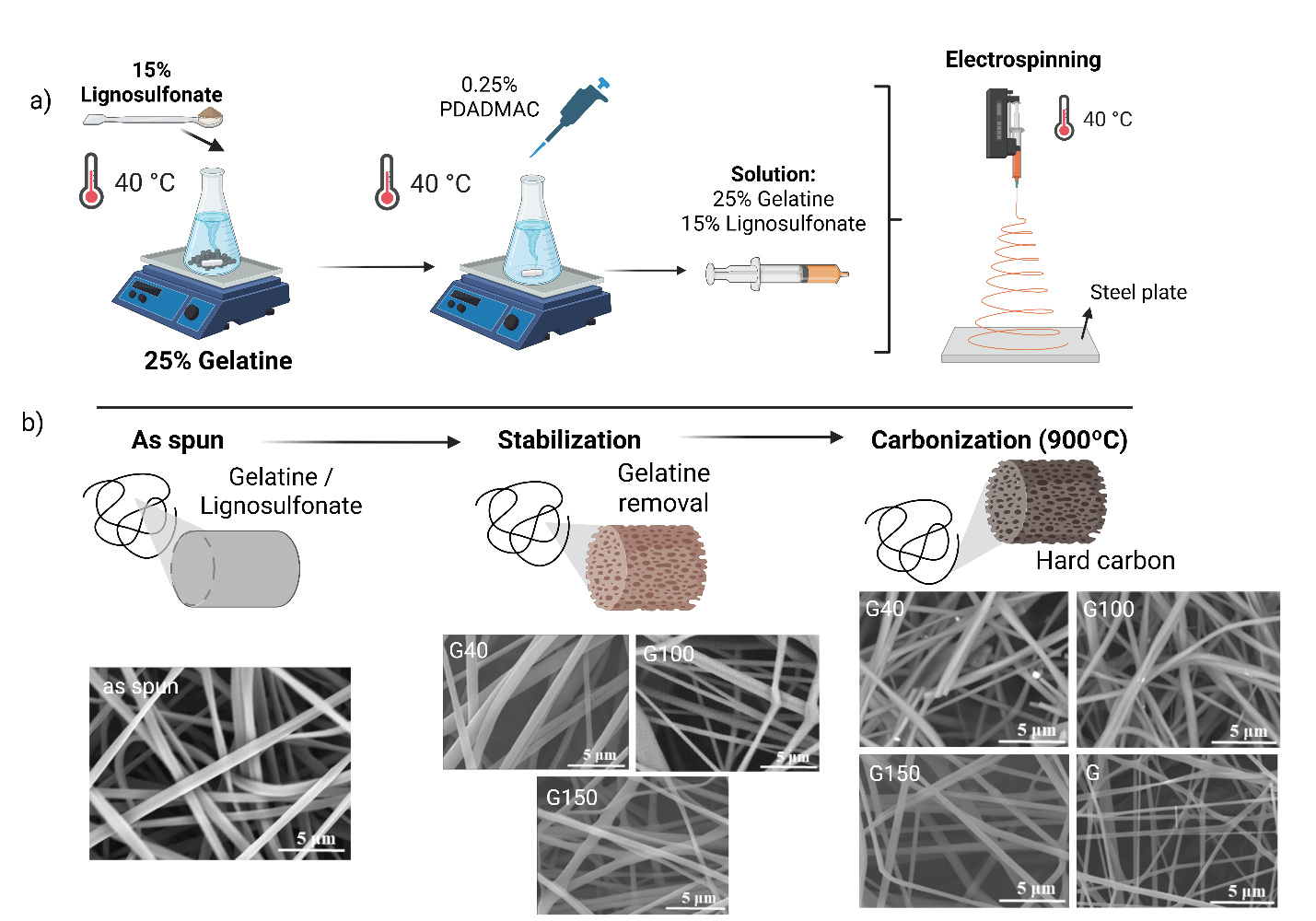
**

**Figure S2. Schematic representation of gelatin/lignosulfonate-derived hard carbon nanofibers workflow. a) Solution preparation and electrospinning; b) Carbon fiber obtention from carbon precursor.**

Post-electrospinning, optimized samples were stabilized using four different protocols, shown in Table S1. The temperature was ramped at 1 °C/min, keeping the isotherm for 1 hour. The last sample was directly carbonized, without any stabilization.

**Table S1. Tested stabilization protocols over the optimized composition.**

| **Sample** | **1^st^ isotherm**  **(for 1 hour)** | **2^nd^ isotherm**  **(for 1 hour)** | **3^rd^ isotherm**  **(for 1 hour)** | **4^th^ isotherm**  **(for 1 hour)** | **5^th^ isotherm**  **(for 1 hour)** | **6^th^ isotherm**  **(for 1 hour)** |
| --- | --- | --- | --- | --- | --- | --- |
| G | No stabilization, direct carbonization | | | | | |
| G40 | 40 °C | 100 °C | 150 °C | 170 °C | 200 °C | 250 °C |
| G100 | 100 °C | 150 °C | 170 °C | 200 °C | 250 °C | - |
| G150 | 150 °C | 170 °C | 200 °C | 250 °C | - | - |

Stabilized lignosulfonate/gelatin nanofibers were carbonized using a tubular furnace heating from room temperature to 900 °C at 10 °C/min under N_2_ flow and at 900 °C for 30 min.

**Characterization of the nanostructured CNFs**

Scanning Electron Microscopy (SEM) was carried out in a Hitachi SU-70 (Hitachi High-Technologies Corporation, Tokyo, Japan) to evaluate the fiber morphology, homogeneity, structural integrity, and changes during the synthesis process. The accelerating voltage during the SEM observation was 10-15 kV. Using ImageJ software over the SEM pictures, the fiber diameter and the Standard Deviation (STD) were calculated. The Horiba LabRAM 1A Raman spectrometer equipped with a 514 nm laser was used to measure Raman spectra of hard carbon after carbonization at room temperature in a backscattering configuration. A silicon sample spectrum was used to calibrate all measurements, and the spectrometer was kept in the same position to ensure accuracy. PANalytical Empyrean instrument equipped with a Cu Kα radiation source (λ = 1.5418 A) was used for X-ray diffraction (XRD). Fourier-transformed infrared spectroscopy (FT-IR) PerkinElmer (Waltham, MA) Spectrum 100 spectrometer with an attenuated total reflectance (ATR) accessory was used on the fibers as spun and after the stabilization. X-ray photoelectron spectroscopy (XPS) analysis was performed on the carbonized samples to see the binding energy using the Kratos AXIS ULTRA spectrometer. *XPS C 1s spectra were deconvoluted using a Gaussian–Lorentzian peak fitting method with the following component ranges: sp² C at 284.4–284.9 eV, sp³ C at 285.0–285.4 eV, C–O at 286.2–286.6 eV, C=O at 287.4–287.9 eV, and O–C=O at 288.8–289.4 eV; FWHM values were restricted to 0.6–1.2 eV for sp²/sp³. Sp² and sp³ hybrid orbitals were assigned to the C=C and C-C components, respectively, as reported in literature [41].”*

Thermogravimetric Analysis (TGA) analysis of the carbon precursors was carried out in a TGA 550 from TA instruments to determine the carbon yield of each case.

**Electrochemical testing**

For the sodium ion battery tests, sodium metal was used as counter electrode and 1M sodium triflate (NaCF_3_SO_3_) dissolved in Bis(2-methoxyethyl) Ether (DEGDME) as electrolyte. Coin-type cells (CR2032) were assembled in an argon-filled glovebox with oxygen and moisture levels of less than 1 ppm. The galvanostatic charge/discharge tests were performed using a NEWARE battery tester at different current densities with a 0.01-2.0 V cutoff voltage window. No slurry was performed for the electrochemical testing, free-standing electrodes of 1cm of diameter were used, hence, the material was used as an electrode directly after carbonization.

**Optimization of the preparation of gelatin fibers**

The electrospinnability of gelatin dissolved into different aqueous-based solutions was tested to obtain the best composition. For that, gelatin solutions using different solvents were prepared, Table S2. The gelatin powder was weighted and then added into vials. This solution was kept under continuous stirring overnight at 40 °C.

**Table S2. Tested compositions for aqueous-based gelatin solutions electrospinning, 50 mL total volume.**

| **Gelatin**  **(g)** | **Alginate**  **(g)** | | **Acetic acid in water (mL)** | **PDADMAC in water (μL)** | **Solid content (%)** |
| --- | --- | --- | --- | --- | --- |
| 7.5 | - | 1 | | - | 15 |
| 10 | - | 1 | | - | 20 |
| 12.5 | - | 1 | | - | 25 |
| 15 | - | 0.5 | | - | 15 |
| 10 | - | 0.5 | | - | 20 |
| 12.5 | - | 0.5 | | - | 25 |
| 10 | 1 | - | | - | 22 |
| 10 | 0.5 | - | | 250 | 21 |
| 10 | 0.5 | - | | 500 | 21 |
| 10 | 0.5 | - | | 2500 | 21 |
| 7.5 | - | - | | 125 | 15 |
| 7.5 | - | - | | 250 | 15 |
| 7.5 | - | - | | 375 | 15 |
| 7.5 | - | - | | 500 | 15 |
| 7.5 | - | - | | 1000 | 15 |
| 10 | - | - | | 125 | 20 |
| 10 | - | - | | 250 | 20 |
| 10 | - | - | | 375 | 20 |
| 10 | - | - | | 500 | 20 |
| 10 | - | - | | 1000 | 20 |
| 12.5 | - | - | | 125 | 25 |
| 12.5 | - | - | | 250 | 25 |
| 12.5 | - | - | | 375 | 25 |
| 12.5 | - | - | | 500 | 25 |
| 12.5 | - | - | | 1000 | 25 |
| 15 | - | - | | 125 | 30 |
| 15 | - | - | | 250 | 30 |
| 15 | - | - | | 375 | 30 |
| 15 | - | - | | 500 | 30 |
| 15 | - | - | | 1000 | 30 |

**Optimization of the preparation of gelatin fibers**

At first, the solutions made from 20% Gelatin were electrospun. Two of the most important parameters when electrospinning are the surface tension and the viscosity of the solution. To electrospun gelatin, one of the most used solvents is acetic acid. In this first experiment we compare the efficacy of dissolving gelatin using different strategies. Acetic acid was used to compare with the new approaches, attending to the key factors that affect the electrospinning of a solution, alginate was used to increase the viscosity of the electrospun solution and surfactant PDADMAC was used to decrease the surface tension of the solution. Figure S2 shows that all of the solutions can respond to the voltage applied and form jets but the solutions that had 2% alginate. The solution with 2% acetic acid presents some beading whereas 0.5% PDADMAC gives homogenous and non-beaded fibers. The alginate solutions were difficult to electrospin and the solutions failed to give stable fibers.

**
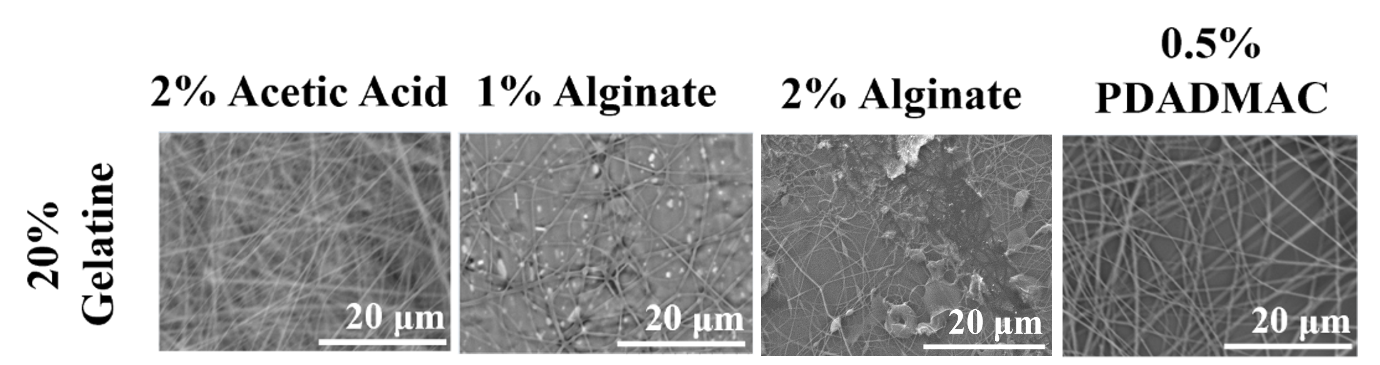
**

**Figure S3: 20% gelatin disolved in an aqueous solution with alginate, acetic acid or PDADMAC to test viability of the electrospun fibers.**

As the use of surfactant improve the electrospinnability, new compositions based on 20% Gelatin and 1% Alginate were tested. This time, 0.5-1-5% PDADMAC was added to the solutions to see if PDADMAC was able to improve the electrospinnability of the gelatin and alginate solutions. Figure S3 shows that stable and non-beaded fibers were obtained when increasing the amount of surfactant added to the solution. Alginate and gelatin dissolved in water on their own is not enough to obtain fibers, but when surfactant is added to it, homogenous fibers can be created. Thus, confirming the idea that the surfactant lowers the surface tension of the solution and can improve the spinnability.

**
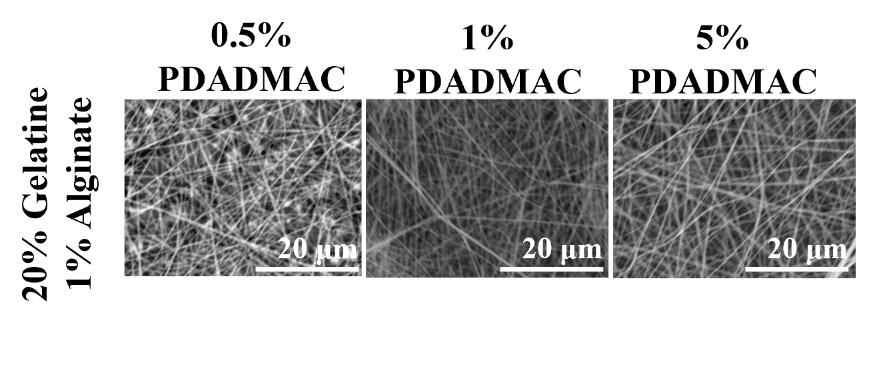
**

**Figure S4: Electrospun fibers using 20% of gelatin and 1% of alginate disolved in water with different percentages of PDADMAC.**

As it occurred, the addition of alginate did not have a big impact on the spinnability of the solutions, but the surfactant did. During the subsequent experiments, different proportions of acetic acid and gelatin were tested to better understand the impact of the acid, Figure S4. Normally, gelatin would be dissolved in high concentration of acetic acid in water, but as the goal was to decrease as much as possible the presence of acids and promote water as the sole solvent, 2% acetic acid in water was chosen as the highest concentration. When increasing the concentration of gelatin from 15% to 25% transitioning from electrospraying to electrospinning. Nonetheless, when decreasing the amount of acid used to dissolve the gelatin, not electrospraying nor electrospinning did happen as the concentration of acid seemed to not play a key role in stabilising the electrospinning process for these compositions.

**
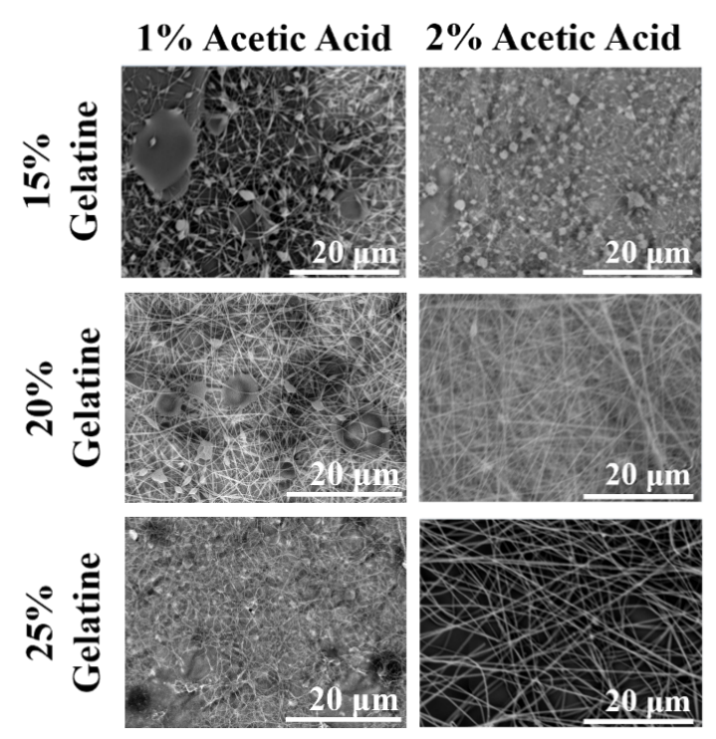
**

**Figure S5: Electrospun fibers of varying percentages of gelatin dissolved in an aqueous solution of 1 or 2% of acetic acid.**

**
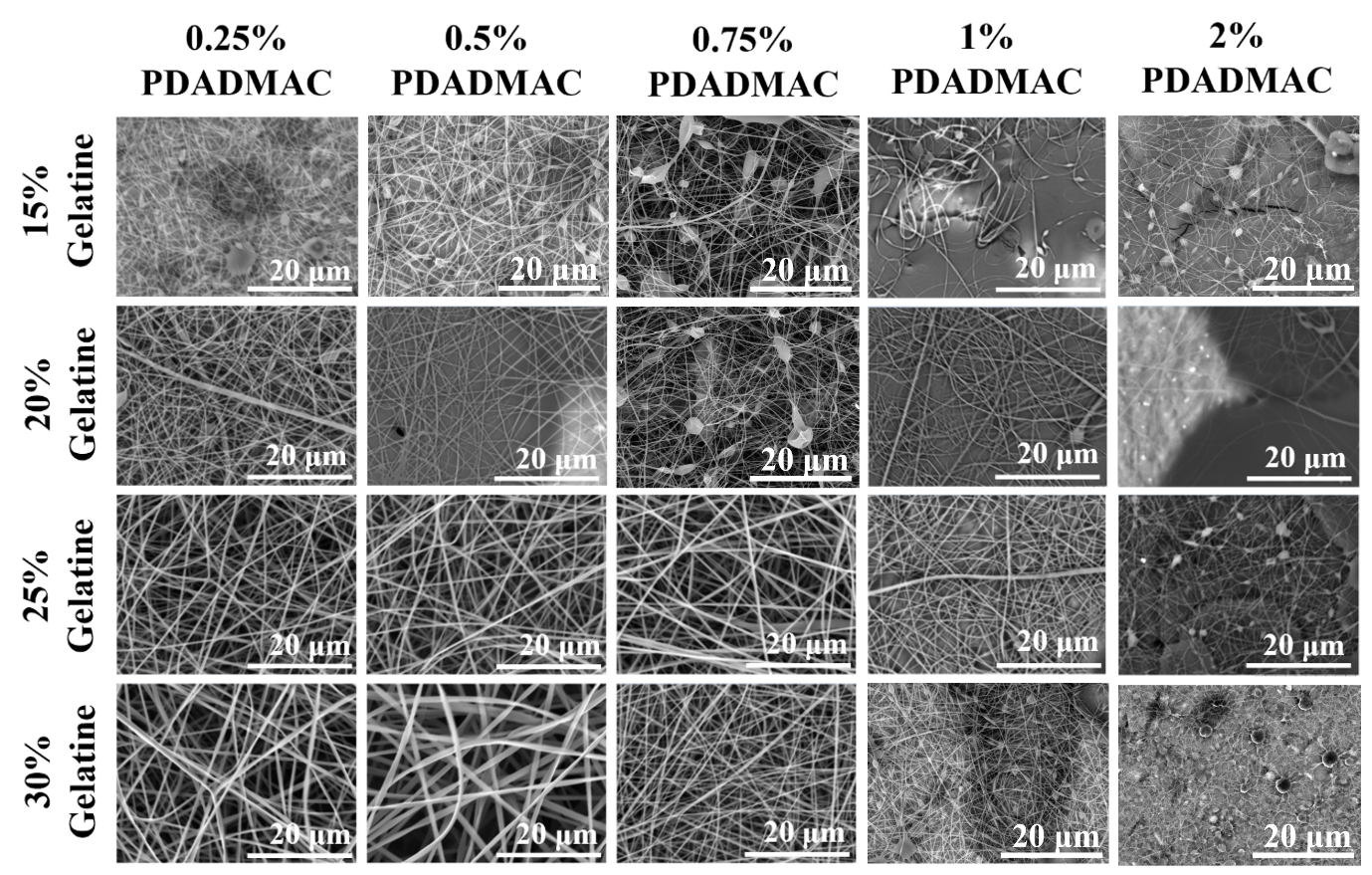
**

**Figure S6. Electrospun fibers of varying percentages of gelatin dissolved in an aqueous solution of varying percentages of PDADMAC.**

Different concentrations of gelatin and PDADMAC were tested to see how they affected one another. Figure S5 shows that the two most stable solutions were 20% and 25% gelatin and that, as we decreased the concentration of surfactant, more stable fibers were obtained. The optimal concentration of PDADMAC was settled to be between 0.25% and 0.75%. PDADMAC is a cationic surfactant. In previous studies concerning the effect of different types of surfactants it was found that the optimal concentration of cationic surfactants to achieve a decrease in the surface tension of water-in-oil emulsions was 0.8-1.2, for anionic surfactants the optimal concentration was 1% and for non-ionic surfactants the optimal concentration was found to be in a range of 0.2%-1%.

**Optimization of the preparation of lignosulfonate/gelatin fibers**

**
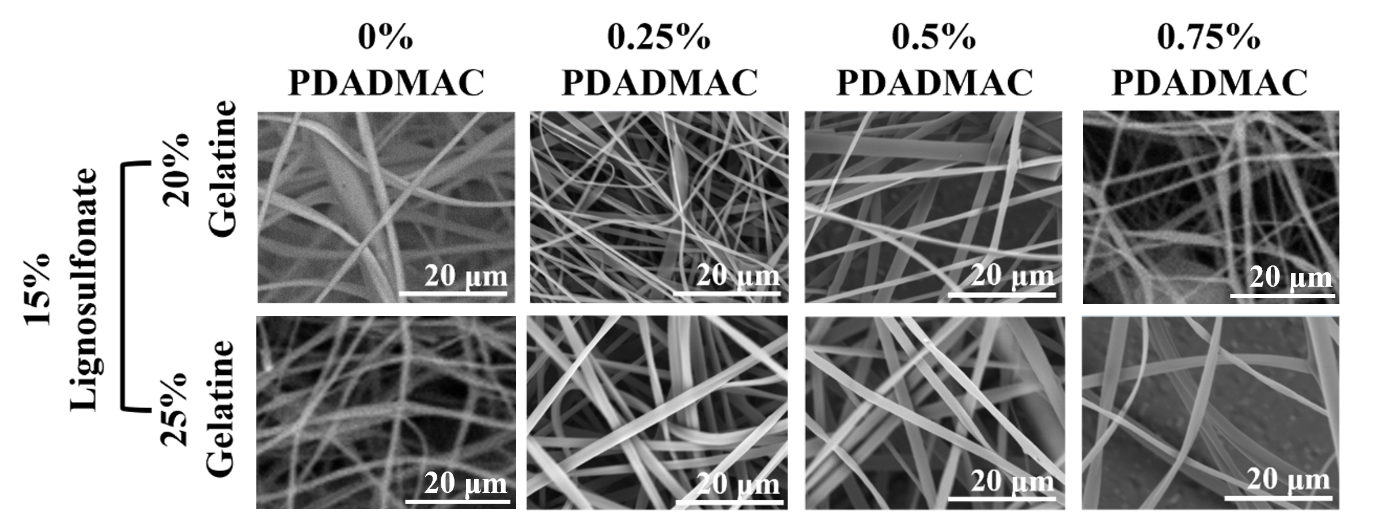
**

**Figure S7. Electrospun fibers of 20 and 25% of gelatin and 15% lignosulfonate dissolved in an aqueous solution of varying percentages of PDADMAC.**

15% of lignosulfonate was added to the solutions of 20% and 25% gelatin with 0, 0.25, 0.5 and 0.75% PDADMAC. There was an improvement of the electrospinning due to the chemical composition of lignosulfonate, that is heavily dopped with sulfuric radicals, hence we can consider that lignosulfonate acts as a polyelectrolyte stabilizing the air-liquid interfaces of the Taylor cone when electrospinning. When no PDADMAC is added the fibers lack homogeneity and it is more difficult to electrospin, avoiding fiber obtention when using the solution of 25% gelatin. The best fibers are obtained when electrospinning 25% gelatin with 15% lignosulfonate, increasing the amount of gelatin while decreasing the amount of PDADMAC improves electrospinning of the solutions, Figure S6.

Figure S7 shows SEM images of the spun fibers, after stabilization at 150 °C and subsequent carbonization. It was noted that the structural integrity of the fibers is only maintained when the concentration of PDADMAC is 0.25%. For 0.5% PDADMAC, the fibers turn into ribbon-like structures. In addition, the 0.75% PDADMAC did not achieve stable structural integrity after the stabilization process. Figure S8 shows SEM images of the same compositions undergoing direct carbonization. The 0.25% PDADMAC concentration shows the best structural integrity, whereas the fibers turn into ribbon-like structures with the 0.50% PDADMAC. This also occurs with the 0.75% PDADMAC as the fibers are not homogeneous and they follow a ribbon-like, broken shape.

**
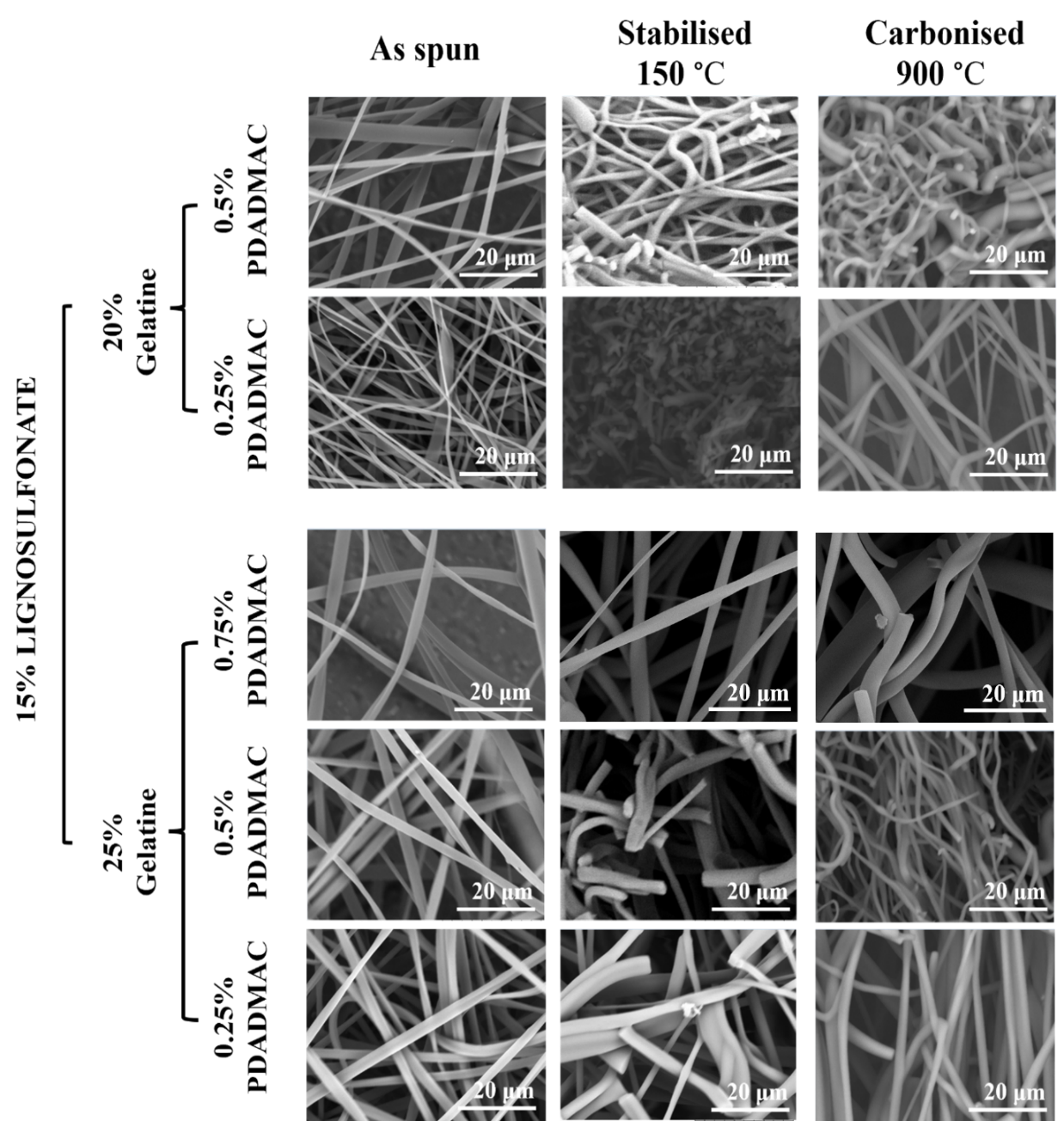
**

**Figure S8. Stabilization protocol tested over the most stable compositions of gelatin/lignosulfonate fibers.**

**
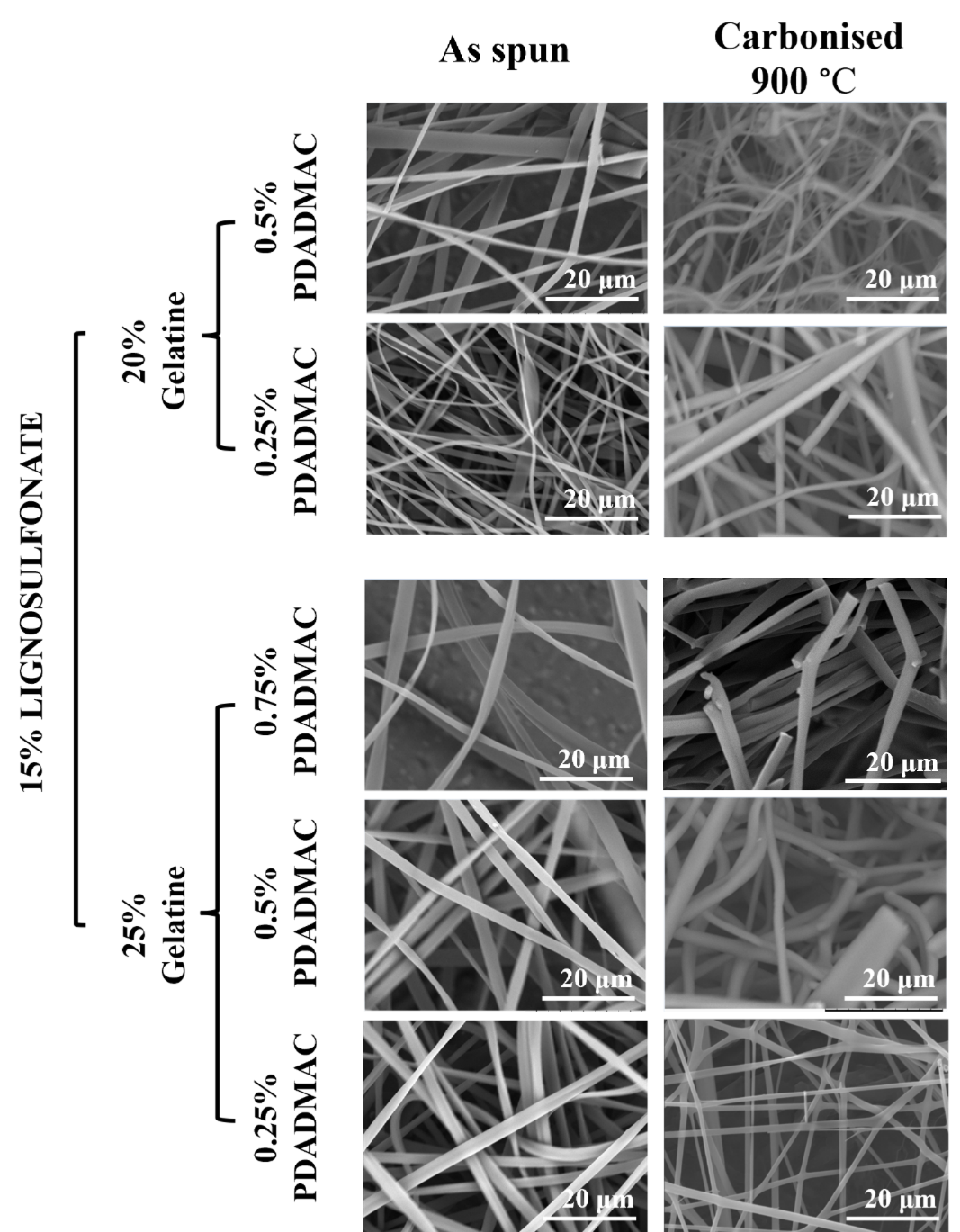
**

**Figure S9. SEM pictures of the fibers after direct carbonization.**

XPS was performed on the carbonized samples to characterize their surface chemistry. The full spectrum acquired shows that the samples are mainly composed of carbon and oxygen, with the stabilized samples having more oxygen concentration, see Figure S9. It is important to highlight that the XPS was able to find traces of Na 1s, N 1s, and Si 2p on all the samples; S 2p in G, G40 and G100; and K 2s in G40 and G100, which backs up the presence of impurities found in the XRD analysis due to the direct carbonization undergone by sample G, Figure 2b

**
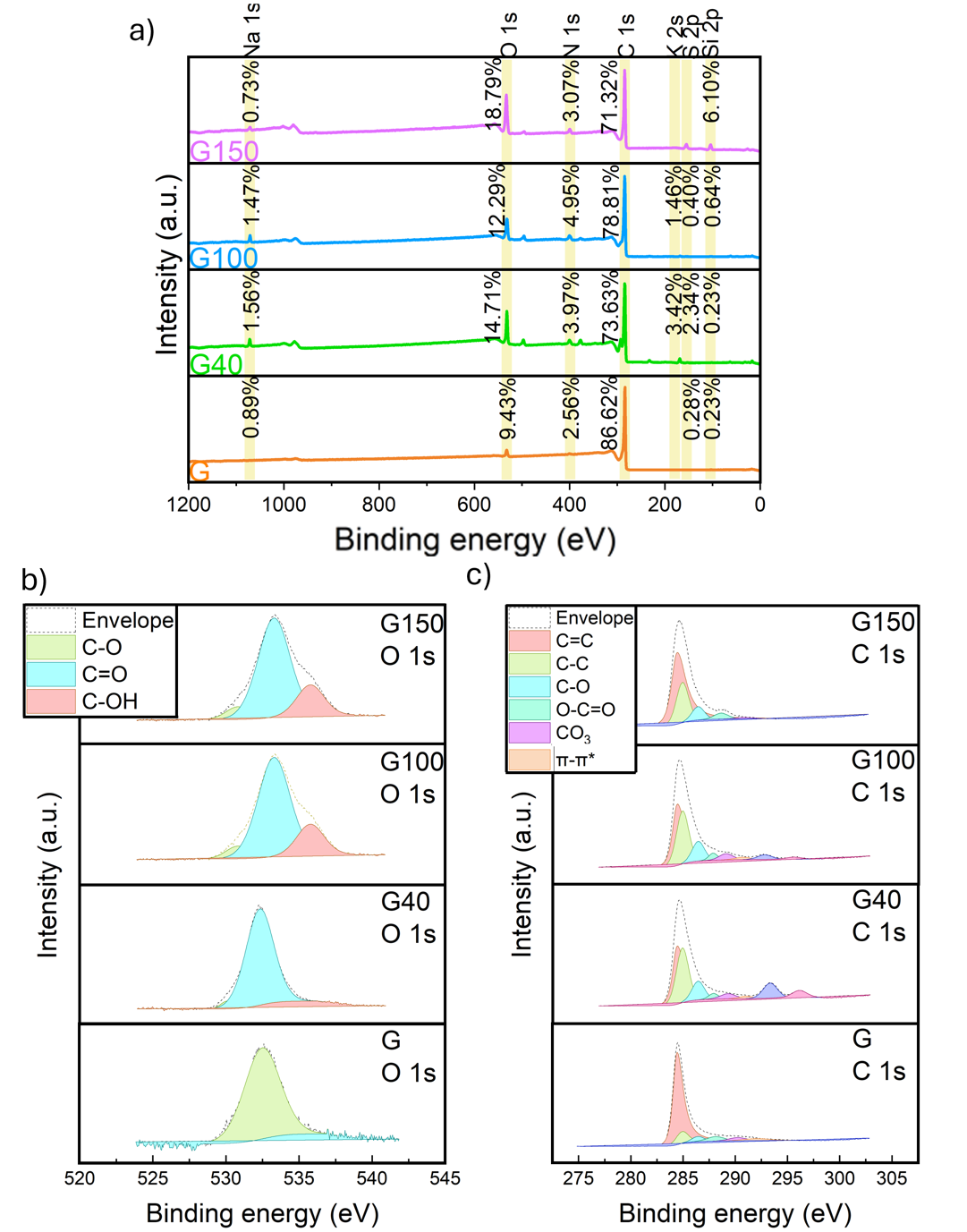
**

**Figure S10. XPS of G150, G100, G40 and G, centered for carbon and oxygen.**

**
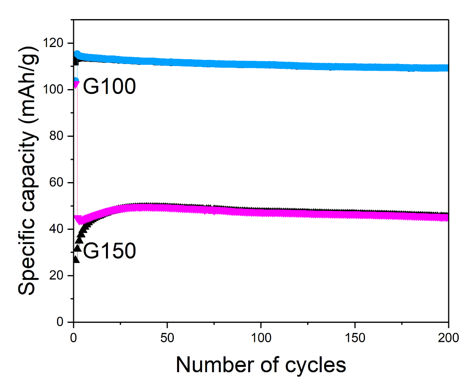
**

**Figure S11. Electrochemical testing of G100 and G150 as SIB anode materials. Cyclic performance of a) G100 5.7 mg/cm^2^ mass loading; and b) G150 5.6 mg/cm^2^ mass loading samples at 100 mA/g.**

**
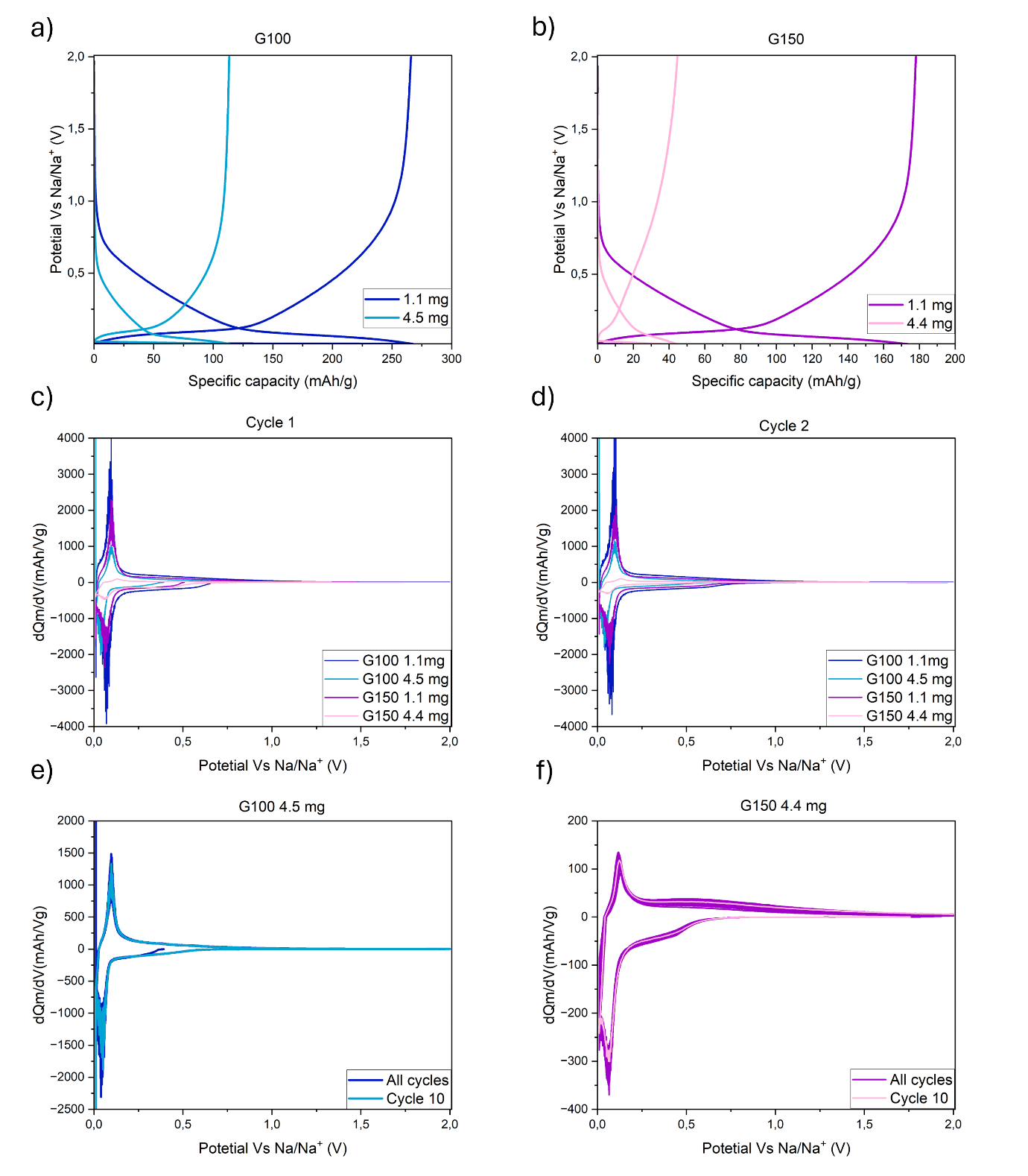
**

**Figure S12. Electrochemical testing of G100 and G150 as SIB anode materials, using different mass loading. a) GCD curves of 10th cycle of G100 using different mass loadings; b) GCD curves of 10th cycle of G150 using different mass loadings. Differential capacity plot of G100 and G150 electrodes (electrodes were cycled at 100 mA g−1 in the potential range of 0.01−2.0 V): c) Cycle 1 and d) Cycle 2; e) G100 5.7 mg/cm^2^ mass loading all cycles and Cycle 10, f) G150 5.6 mg/cm^2^ mass loading all cycles and Cycle 10.**

When increasing the mass loading there is a less intense peak <0.1 V which relates to a lower plateau capacity, Figure S11c and S11d. The reduction peak of 0.2 - 0.0 V corresponds to the insertion of sodium ions into the electrode during the sodiation process and the oxidation peak of 2.0−0.2 V is due to their extraction during the desodiation process. The sloped region between 2.0 and 0.2 V is ascribed to the insertion of sodium ions into the edges or pores of the fibers. When all the cycles are plotted together, the voltage profiles almost overlap, indicating that the sodiation and desodiation processes of fibers possess good reversibility during cycling. In addition, because of the absence of a peak shift, we know there is no buildup of overpotential, and the diffusion of the sodium ions is fluent and stable over time.

However, when the higher mass loading samples is analyzed (Figure S11a-b) there’s more contribution from the slope behavior, this is due to the resistivity effect of increasing mass loading. A way to decrease the resistivity effect is by decreasing the current density at which the batteries are cycled. Figure S12 shows the results obtained when the batteries are cycled at 50 mA/g instead of 100 mA/g. When decreasing the current intensity, sample G100 shows an average specific capacity of 182.7 mAh/g over 20 cycles, with a CE of 99.64%. In the case of G150, the average specific capacity is 130.5 mAh/g over 20 cycles, with a CE of 99.89%.

**
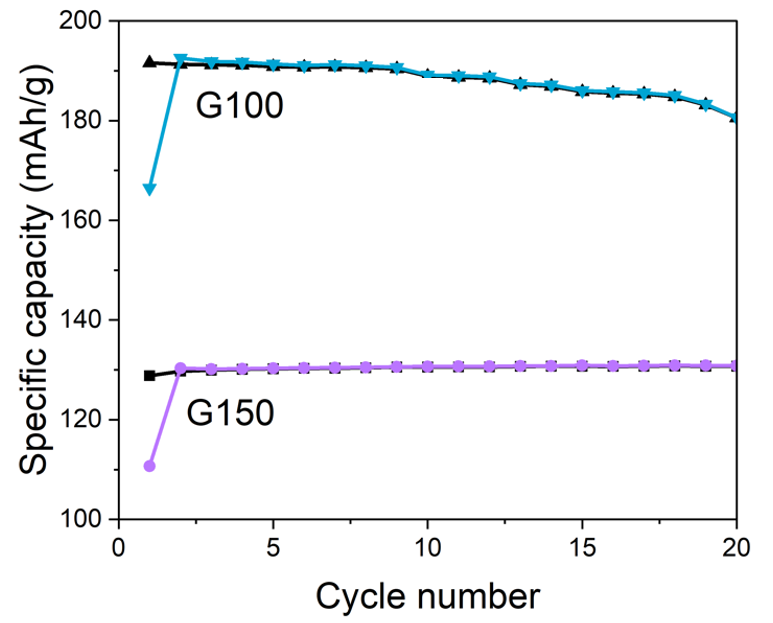
**

**Figure S13. Electrochemical test for G100 5.7 mg/cm^2^ mass loading and G150 5.6 mg/cm^2^ mass loading as SIB anode material. Cyclic performance at 50 mA/g.**

**Section 1. Density Functional Theory (DFT) Calculations**

All quantum-chemical calculations were performed using Gaussian 16, Revision C.01[44]. Geometry optimisations and energy evaluations were conducted using DFT with the B3LYP[45] functional and the 6-31+G(d,p)[46] basis set for all atoms. Sodium was treated as Na⁺ throughout. Basis-set superposition error (BSSE)[47] was corrected using the counterpoise method as implemented in Gaussian.

To model the distinct carbon environments observed experimentally in G100 and G150, three coronene-based structures were constructed:

1. Model 1: Pristine Coronene (sp² graphitic)
2. Model 2: OH-Functionalised Coronene (mild edge sp³/oxygenated defect)
3. Model 3: Vacancy-Type Defective Coronene (sp³-rich cavity)

For each model, the computational workflow consisted of the following steps.

**Geometry Optimisation of Carbon Models**

Each coronene-derived structure was optimised in the gas phase with no symmetry constraints applied. All structures were confirmed to have converged through Gaussian’s default geometry-convergence criteria. The optimised structures from this step were used as the *receptor frameworks* for subsequent Na⁺ binding calculations.

**Geometry Optimisation of Na⁺–Coronene Complexes**

For each model, a Na⁺ ion was placed above the surface at the most chemically reasonable adsorption site:

1. Model 1: above the central aromatic hollow site
2. Model 2: near the OH-functionalised edge
3. Model 3: partially within the vacancy-rich cavity

Each Na⁺-coronene complex was then fully re-optimised in the gas phase using. The ion and carbon framework were allowed to relax without constraints. Final structures were visually inspected to confirm retention of physically meaningful binding motifs (cation–π interactions, cavity stabilisation, etc.). These optimised complexes were used as inputs for the counterpoise calculations.

**Basis-set superposition error (BSSE)-Corrected Single-Point Counterpoise Calculations**

To obtain accurate Na⁺ binding energies, a single-point calculation using the counterpoise correction was performed on each optimised Na⁺–coronene complex:

Gaussian fragment definitions were:

1. Fragment 1: coronene-based carbon framework
2. Fragment 2: Na⁺

The BSSE-corrected Na⁺ binding energy was computed as:

$E_{\text{bind}}=E_{\text{complex}}-(E_{\text{Na}^{+}}^{*}+E_{\text{coronene}}^{*})$ Eqn. S1

where the *starred* terms denote monomer energies computed in the full dimer basis. This approach ensures that differences in Na⁺ binding strength between sp²-rich and sp³-rich surfaces arise solely from the surface chemistry, not from basis-set artefacts.

**Table S3. Summary of Final BSSE-Corrected Binding Energies**

| Model | Description | Binding Energy (kcal/mol) |
| --- | --- | --- |
| **1** | Pristine coronene (sp²) | **–41.06** |
| **2** | OH-functionalised coronene (mild sp³) | **–40.87** |
| **3** | Vacancy-type defective coronene (sp³-rich cavity) | **–122.89** |

The pristine and OH-functionalised surfaces bind Na⁺ with similar and relatively weak interaction energies (~–41 kcal/mol). In contrast, the high-defect, sp³-rich cavity stabilises Na⁺ nearly three times more strongly. These DFT results correlate directly with the XPS trends:

1. G150 (sp³/sp² = 0.39): predominantly graphitic modelled by pristine coronene leads to weak Na⁺ binding.

2. G100 (sp³/sp² = 0.71): defect-rich, partially sp³ hybridised modelled by defective cavity leads to strong Na⁺ binding.

**REFERENCES**

1. Fan, X., et al., *Research progress on hard carbon materials in advanced sodium-ion batteries.* Energy Storage Materials, 2024. **69**: p. 103386.

2. Nita, C., et al., *Hard carbon derived from coconut shells, walnut shells, and corn silk biomass waste exhibiting high capacity for Na-ion batteries.* Journal of Energy Chemistry, 2021. **58**: p. 207-218.

3. Long, S.-Y., et al., *Study on the lignin-derived sp2–sp3 hybrid hard carbon materials and the feasibility for industrial production.* Scientific Reports, 2024. **14**(1): p. 5091.

4. Dou, X., et al., *Hard carbons for sodium-ion batteries: Structure, analysis, sustainability, and electrochemistry.* Materials Today, 2019. **23**: p. 87-104.

5. Lesiak, B., et al., *C sp2/sp3 hybridisations in carbon nanomaterials – XPS and (X)AES study.* Applied Surface Science, 2018. **452**: p. 223-231.

6. Marie-Luce, T. and P. Valérie, *Spatial organization of the sp2-hybridized carbon atoms and electronic density of states of hydrogenated amorphous carbon films.* Carbon, 2002. **40**(8): p. 1153-1166.

7. Moon, H., et al., *Bio-Waste-Derived Hard Carbon Anodes Through a Sustainable and Cost-Effective Synthesis Process for Sodium-Ion Batteries.* ChemSusChem, 2023. **16**(1): p. e202201713.

8. Liu, Q., L. Luo, and L. Zheng, *Lignins: Biosynthesis and Biological Functions in Plants.* Int J Mol Sci, 2018. **19**(2).

9. Das, O., et al., *Natural and industrial wastes for sustainable and renewable polymer composites.* Renewable and Sustainable Energy Reviews, 2022. **158**.

10. Zhang, C., *Lignocellulosic ethanol: technology and economics.* Alcohol fuels-current technologies and future prospect, 2019.

11. Ayyachamy, M., et al., *Lignin: untapped biopolymers in biomass conversion technologies.* Biomass Conversion and Biorefinery, 2013. **3**(3): p. 255-269.

12. Shepa, I., E. Múdra, and J. Dusza, *Electrospinning through the prism of time.* Materials Today Chemistry, 2021.

13. Yang, X., et al., *Structural design toward functional materials by electrospinning: A review.* e-Polymers, 2020. **20**(1): p. 682-712.

14. Xue, J., et al., *Electrospinning and Electrospun Nanofibers: Methods, Materials, and Applications.* Chem Rev, 2019. **119**(8): p. 5298-5415.

15. Nicole, A., et al., *Recent advances in electrospinning of nanofibers from bio-based carbohydrate polymers and their applications.* Trends in Food Science & Technology, 2022. **120**: p. 308-324.

16. Li, Y., et al., *Developments of Advanced Electrospinning Techniques: A Critical Review.* Advanced Materials Technologies, 2021. **6**(11): p. 2100410.

17. Zhuang, X., et al., *Electrospun chitosan/gelatin nanofibers containing silver nanoparticles.* Carbohydrate Polymers, 2010. **82**(2): p. 524-527.

18. Bazmandeh, A.Z., et al., *Dual spinneret electrospun nanofibrous/gel structure of chitosan-gelatin/chitosan-hyaluronic acid as a wound dressing: In-vitro and in-vivo studies.* International Journal of Biological Macromolecules, 2020. **162**: p. 359-373.

19. Gulzar, S., et al., *Electrospinning of gelatin/chitosan nanofibers incorporated with tannic acid and chitooligosaccharides on polylactic acid film: Characteristics and bioactivities.* Food Hydrocolloids, 2022. **133**: p. 107916.

20. Ahmadi, S., et al., *Cinnamon extract loaded electrospun chitosan/gelatin membrane with antibacterial activity.* International Journal of Biological Macromolecules, 2021. **173**: p. 580-590.

21. Ferreira, C.A.M., et al., *Multifunctional Gelatin/Chitosan Electrospun Wound Dressing Dopped with Undaria pinnatifida Phlorotannin-Enriched Extract for Skin Regeneration.* Pharmaceutics, 2021. **13**(12).

22. Hajinasrollah, K., S. Habibi, and H. Nazockdast, *Fabrication of gelatin–chitosan–gum tragacanth with thermal annealing cross-linking strategy.* Journal of Engineered Fibers and Fabrics, 2019. **14**.

23. Bakhsheshi-Rad, H.R., et al., *In vitro and in vivo evaluation of chitosan-alginate/gentamicin wound dressing nanofibrous with high antibacterial performance.* Polymer Testing, 2020. **82**: p. 106298.

24. Pan, L., et al., *High-performance Porous Electrodes for Flow Batteries: Improvements of Specific Surface Areas and Reaction Kinetics.* ChemElectroChem, 2024. **11**(21): p. e202400460.

25. Boeriu, C.G., et al., *Characterisation of structure-dependent functional properties of lignin with infrared spectroscopy.* Industrial Crops and Products, 2004. **20**(2): p. 205-218.

26. Seydibeyoğlu, M.Ö., *A Novel Partially Biobased PAN-Lignin Blend as a Potential Carbon Fiber Precursor.* BioMed Research International, 2012. **2012**(1): p. 598324.

27. Ferrari, A.C., *Raman spectroscopy of graphene and graphite: Disorder, electron–phonon coupling, doping and nonadiabatic effects.* Solid State Communications, 2007. **143**(1): p. 47-57.

28. Cançado, L.G., et al., *Science and Metrology of defects in graphene using Raman Spectroscopy.* Carbon, 2024. **220**: p. 118801.

29. Eom, Y., et al., *Structure evolution mechanism of highly ordered graphite during carbonization of cellulose nanocrystals.* Carbon, 2019. **150**: p. 142-152.

30. Eraghi Kazzaz, A. and P. Fatehi, *Technical lignin and its potential modification routes: A mini-review.* Industrial Crops and Products, 2020. **154**: p. 112732.

31. Ding, B., et al., *Redox-active ligands: Recent advances towards their incorporation into coordination polymers and metal-organic frameworks.* Coordination Chemistry Reviews, 2021. **439**: p. 213891.

32. Qu, G., Z. Ma, and T. Jia, *Influence of Hydroxyl Groups on the Oxidative Reaction Characteristics of Active Groups in Lignite at Room Temperature.* ACS Omega, 2024. **9**(14): p. 16237-16248.

33. Tan, J., et al., *Structural insights into solid electrolyte interphase (SEI) on lithium metal anode: From design strategies to the stability evaluation.* Materials Today, 2023. **69**: p. 287-332.

34. Umada, N., et al., *Oriented sp2 bonded carbon structure of hydrogenated amorphous carbon films.* Diamond and Related Materials, 2023. **131**: p. 109533.

35. Lu, H., et al., *Exploring Sodium-Ion Storage Mechanism in Hard Carbons with Different Microstructure Prepared by Ball-Milling Method.* Small, 2018. **14**(39): p. 1802694.

36. Wang, F., T. Zhang, and F. Ran, *Insights into sodium-ion batteries through plateau and slope regions in cyclic voltammetry by tailoring bacterial cellulose precursors.* Electrochimica Acta, 2023. **441**: p. 141770.

37. Cao, Y., et al., *Sodium Ion Insertion in Hollow Carbon Nanowires for Battery Applications.* Nano Letters, 2012. **12**(7): p. 3783-3787.

1. Fan, X., et al., *Research progress on hard carbon materials in advanced sodium-ion batteries.* Energy Storage Materials, 2024. **69**: p. 103386.

2. Nita, C., et al., *Hard carbon derived from coconut shells, walnut shells, and corn silk biomass waste exhibiting high capacity for Na-ion batteries.* Journal of Energy Chemistry, 2021. **58**: p. 207-218.

3. Long, S.-Y., et al., *Study on the lignin-derived sp2–sp3 hybrid hard carbon materials and the feasibility for industrial production.* Scientific Reports, 2024. **14**(1): p. 5091.

4. Dou, X., et al., *Hard carbons for sodium-ion batteries: Structure, analysis, sustainability, and electrochemistry.* Materials Today, 2019. **23**: p. 87-104.

5. Lesiak, B., et al., *C sp2/sp3 hybridisations in carbon nanomaterials – XPS and (X)AES study.* Applied Surface Science, 2018. **452**: p. 223-231.

6. Marie-Luce, T. and P. Valérie, *Spatial organization of the sp2-hybridized carbon atoms and electronic density of states of hydrogenated amorphous carbon films.* Carbon, 2002. **40**(8): p. 1153-1166.

7. Moon, H., et al., *Bio-Waste-Derived Hard Carbon Anodes Through a Sustainable and Cost-Effective Synthesis Process for Sodium-Ion Batteries.* ChemSusChem, 2023. **16**(1): p. e202201713.

8. Liu, Q., L. Luo, and L. Zheng, *Lignins: Biosynthesis and Biological Functions in Plants.* Int J Mol Sci, 2018. **19**(2).

9. Das, O., et al., *Natural and industrial wastes for sustainable and renewable polymer composites.* Renewable and Sustainable Energy Reviews, 2022. **158**.

10. Zhang, C., *Lignocellulosic ethanol: technology and economics.* Alcohol fuels-current technologies and future prospect, 2019.

11. Ayyachamy, M., et al., *Lignin: untapped biopolymers in biomass conversion technologies.* Biomass Conversion and Biorefinery, 2013. **3**(3): p. 255-269.

12. Shepa, I., E. Múdra, and J. Dusza, *Electrospinning through the prism of time.* Materials Today Chemistry, 2021.

13. Yang, X., et al., *Structural design toward functional materials by electrospinning: A review.* e-Polymers, 2020. **20**(1): p. 682-712.

14. Xue, J., et al., *Electrospinning and Electrospun Nanofibers: Methods, Materials, and Applications.* Chem Rev, 2019. **119**(8): p. 5298-5415.

15. Nicole, A., et al., *Recent advances in electrospinning of nanofibers from bio-based carbohydrate polymers and their applications.* Trends in Food Science & Technology, 2022. **120**: p. 308-324.

16. Li, Y., et al., *Developments of Advanced Electrospinning Techniques: A Critical Review.* Advanced Materials Technologies, 2021. **6**(11): p. 2100410.

17. Zhuang, X., et al., *Electrospun chitosan/gelatin nanofibers containing silver nanoparticles.* Carbohydrate Polymers, 2010. **82**(2): p. 524-527.

18. Bazmandeh, A.Z., et al., *Dual spinneret electrospun nanofibrous/gel structure of chitosan-gelatin/chitosan-hyaluronic acid as a wound dressing: In-vitro and in-vivo studies.* International Journal of Biological Macromolecules, 2020. **162**: p. 359-373.

19. Gulzar, S., et al., *Electrospinning of gelatin/chitosan nanofibers incorporated with tannic acid and chitooligosaccharides on polylactic acid film: Characteristics and bioactivities.* Food Hydrocolloids, 2022. **133**: p. 107916.

20. Ahmadi, S., et al., *Cinnamon extract loaded electrospun chitosan/gelatin membrane with antibacterial activity.* International Journal of Biological Macromolecules, 2021. **173**: p. 580-590.

21. Ferreira, C.A.M., et al., *Multifunctional Gelatin/Chitosan Electrospun Wound Dressing Dopped with Undaria pinnatifida Phlorotannin-Enriched Extract for Skin Regeneration.* Pharmaceutics, 2021. **13**(12).

22. Hajinasrollah, K., S. Habibi, and H. Nazockdast, *Fabrication of gelatin–chitosan–gum tragacanth with thermal annealing cross-linking strategy.* Journal of Engineered Fibers and Fabrics, 2019. **14**.

23. Bakhsheshi-Rad, H.R., et al., *In vitro and in vivo evaluation of chitosan-alginate/gentamicin wound dressing nanofibrous with high antibacterial performance.* Polymer Testing, 2020. **82**: p. 106298.

24. Pan, L., et al., *High-performance Porous Electrodes for Flow Batteries: Improvements of Specific Surface Areas and Reaction Kinetics.* ChemElectroChem, 2024. **11**(21): p. e202400460.

25. Boeriu, C.G., et al., *Characterisation of structure-dependent functional properties of lignin with infrared spectroscopy.* Industrial Crops and Products, 2004. **20**(2): p. 205-218.

26. Seydibeyoğlu, M.Ö., *A Novel Partially Biobased PAN-Lignin Blend as a Potential Carbon Fiber Precursor.* BioMed Research International, 2012. **2012**(1): p. 598324.

27. Ferrari, A.C., *Raman spectroscopy of graphene and graphite: Disorder, electron–phonon coupling, doping and nonadiabatic effects.* Solid State Communications, 2007. **143**(1): p. 47-57.

28. Cançado, L.G., et al., *Science and Metrology of defects in graphene using Raman Spectroscopy.* Carbon, 2024. **220**: p. 118801.

29. Eom, Y., et al., *Structure evolution mechanism of highly ordered graphite during carbonization of cellulose nanocrystals.* Carbon, 2019. **150**: p. 142-152.

30. Eraghi Kazzaz, A. and P. Fatehi, *Technical lignin and its potential modification routes: A mini-review.* Industrial Crops and Products, 2020. **154**: p. 112732.

31. Ding, B., et al., *Redox-active ligands: Recent advances towards their incorporation into coordination polymers and metal-organic frameworks.* Coordination Chemistry Reviews, 2021. **439**: p. 213891.

32. Qu, G., Z. Ma, and T. Jia, *Influence of Hydroxyl Groups on the Oxidative Reaction Characteristics of Active Groups in Lignite at Room Temperature.* ACS Omega, 2024. **9**(14): p. 16237-16248.

33. Shafiee, F.N., et al., *Recent progress on hard carbon and other anode materials for sodium-ion batteries.* Heliyon, 2024. **10**(8): p. e29512.

34. Mu-Seong, L., et al., *Partially graphitic structure-assisted hard carbon derived from lignin for sodium-ion battery anodes.* Energy Materials, 2025. **5**(9): p. 500104.

35. Chen, B., et al., *Lignin molecular sieving engineering enables high-plateau-capacity hard carbon anodes for sodium-ion batteries.* Green Chemistry, 2024. **26**(13): p. 7919-7930.

36. Wang, X., et al., *A Generic Si-Doped Strategy for Hard Carbon Derived from Wuliangye Distillers’ Grains to Achieve High-Performance Sodium Ion Batteries.* ACS Applied Materials & Interfaces, 2025. **17**(8): p. 12004-12013.

37. Lu, B., et al., *Hard-Carbon Negative Electrodes from Biomasses for Sodium-Ion Batteries.* Molecules, 2023. **28**(10).

38. Zhang, Y., et al., *Honeycomb-like Hard Carbon Derived from Pine Pollen as High-Performance Anode Material for Sodium-Ion Batteries.* ACS Applied Materials & Interfaces, 2018. **10**(49): p. 42796-42803.

39. Hong, K.-l., et al., *Biomass derived hard carbon used as a high performance anode material for sodium ion batteries.* Journal of Materials Chemistry A, 2014. **2**(32): p. 12733-12738.

40. Tan, J., et al., *Structural insights into solid electrolyte interphase (SEI) on lithium metal anode: From design strategies to the stability evaluation.* Materials Today, 2023. **69**: p. 287-332.

41. Umada, N., et al., *Oriented sp2 bonded carbon structure of hydrogenated amorphous carbon films.* Diamond and Related Materials, 2023. **131**: p. 109533.

42. Lu, H., et al., *Exploring Sodium-Ion Storage Mechanism in Hard Carbons with Different Microstructure Prepared by Ball-Milling Method.* Small, 2018. **14**(39): p. 1802694.

43. Wang, F., T. Zhang, and F. Ran, *Insights into sodium-ion batteries through plateau and slope regions in cyclic voltammetry by tailoring bacterial cellulose precursors.* Electrochimica Acta, 2023. **441**: p. 141770.

44. Frisch, M.J., et al., *Gaussian 16 Rev. C.01*. 2016: Wallingford, CT.

45. Lee, C., W. Yang, and R.G. Parr, *Development of the Colle-Salvetti correlation-energy formula into a functional of the electron density.* Phys Rev B Condens Matter, 1988. **37**(2): p. 785-789.

46. Hehre, W.J., R. Ditchfield, and J.A. Pople, *Self—consistent molecular orbital methods. XII. Further extensions of Gaussian—type basis sets for use in molecular orbital studies of organic molecules.* The Journal of Chemical Physics, 1972. **56**(5): p. 2257-2261.

47. Richard, R.M., B.W. Bakr, and C.D. Sherrill, *Understanding the Many-Body Basis Set Superposition Error: Beyond Boys and Bernardi.* J Chem Theory Comput, 2018. **14**(5): p. 2386-2400.

48. Gaur, P., et al., *Molecularly Engineered Memristors for Reconfigurable Neuromorphic Functionalities.* Adv Mater, 2025: p. e09143.

49. Cao, Y., et al., *Sodium Ion Insertion in Hollow Carbon Nanowires for Battery Applications.* Nano Letters, 2012. **12**(7): p. 3783-3787.
